# Comparative genomics of *Bradyrhizobium japonicum* CPAC 15 and *Bradyrhizobium diazoefficiens* CPAC 7: elite model strains for understanding symbiotic performance with soybean

**DOI:** 10.1186/1471-2164-15-420

**Published:** 2014-06-03

**Authors:** Arthur Fernandes Siqueira, Ernesto Ormeño-Orrillo, Rangel Celso Souza, Elisete Pains Rodrigues, Luiz Gonzaga Paula Almeida, Fernando Gomes Barcellos, Jesiane Stefânia Silva Batista, Andre Shigueyoshi Nakatani, Esperanza Martínez-Romero, Ana Tereza Ribeiro Vasconcelos, Mariangela Hungria

**Affiliations:** Department Biochemistry and Biotechnology, Universidade Estadual de Londrina (UEL), C.P. 60001, Londrina, PR 86051-990 Brazil; Embrapa Soja, C.P. 231, Londrina, PR 86001-970 Brazil; Centro de Ciencias Genómicas, Universidad Nacional Autónoma de México, Cuernavaca, Morelos Mexico; Laboratório Nacional de Computação Científica, Rua Getúlio Vargas 333, Petrópolis, RJ 25651-071 Brazil; UEL, Depto. General Biology, C.P. 60001, Londrina, PR 86051-990 Brazil; Department Structural, Molecular and Genetic Biology, Universidade Estadual de Ponta Grossa (UEPG), Av. General Carlos Cavalcanti 4748, Ponta Grossa, PR 84030-900 Brazil

**Keywords:** Symbiosis, Nodulation, Nitrogen fixation, Competitiveness, Secretion systems, Horizontal gene transfer, Membrane transporters, Surface polysaccharides, Secondary metabolism, Phytohormone synthesis

## Abstract

**Background:**

The soybean-*Bradyrhizobium* symbiosis can be highly efficient in fixing nitrogen, but few genomic sequences of elite inoculant strains are available. Here we contribute with information on the genomes of two commercial strains that are broadly applied to soybean crops in the tropics. *B. japonicum* CPAC 15 (=SEMIA 5079) is outstanding in its saprophytic capacity and competitiveness, whereas *B. diazoefficiens* CPAC 7 (=SEMIA 5080) is known for its high efficiency in fixing nitrogen. Both are well adapted to tropical soils. The genomes of CPAC 15 and CPAC 7 were compared to each other and also to those of *B. japonicum* USDA 6^T^ and *B. diazoefficiens* USDA 110^T^.

**Results:**

Differences in genome size were found between species, with *B. japonicum* having larger genomes than *B. diazoefficiens*. Although most of the four genomes were syntenic, genome rearrangements within and between species were observed, including events in the symbiosis island. In addition to the symbiotic region, several genomic islands were identified. Altogether, these features must confer high genomic plasticity that might explain adaptation and differences in symbiotic performance. It was not possible to attribute known functions to half of the predicted genes. About 10% of the genomes was composed of exclusive genes of each strain, but up to 98% of them were of unknown function or coded for mobile genetic elements. In CPAC 15, more genes were associated with secondary metabolites, nutrient transport, iron-acquisition and IAA metabolism, potentially correlated with higher saprophytic capacity and competitiveness than seen with CPAC 7. In CPAC 7, more genes were related to the metabolism of amino acids and hydrogen uptake, potentially correlated with higher efficiency of nitrogen fixation than seen with CPAC 15.

**Conclusions:**

Several differences and similarities detected between the two elite soybean-inoculant strains and between the two species of *Bradyrhizobium* provide new insights into adaptation to tropical soils, efficiency of N_2_ fixation, nodulation and competitiveness.

**Electronic supplementary material:**

The online version of this article (doi: 10.1186/1471-2164-15-420) contains supplementary material, which is available to authorized users.

## Background

Soybean [*Glycine max* (L.) Merr.] is the most important legume cropped worldwide, and the expansion of this crop since the middle of the 20^th^ century—particularly in tropical areas with soils deficient in nitrogen (N)—is attributable to its high capacity in fixing atmospheric nitrogen (N_2_) in symbioses with *Bradyrhizobium*[1]. However, despite the economic and environment importance of this crop, few genomic studies have been reported on elite *Bradyrhizobium* strains. This represents an important opportunity to understand features correlated with high efficiency of nitrogen fixation with the legume. A pioneering genomic study was performed with *B. japonicum* strain USDA 110^T^[2], reclassified now as the type strain of *B. diazoefficiens*[3] and used in commercial inoculants in the United States, in African and other countries [1]. This research highlighted intriguing information about the symbiosis island and high numbers of transposase-encoding and unknown genes [2]. Since then, the genome of *B. japonicum* USDA 6^T^[4] was presented and emphasized evolutionary aspects, possibly because the strain is not outstanding in fixing N_2_. A few other draft genomes of soybean *Bradyrhizobium* strains are now available, but their descriptions also highlighted phylogenetic aspects (e.g. [5]). Altogether, the results clearly point out that other genomes, with an emphasis on elite inoculant strains, still have to be sequenced to get a better understanding of the molecular basis of the “perfect symbiosis” involving soybean and *Bradyrhizobium*[1].

Brazil is the second largest producer of soybean, and is expected to become the largest very soon. Biological fixation of N_2_ has always been a priority in the Brazilian production system. Research programs have focused both on *Bradyrhizobium*-strain selection and identification of plant genotypes of superior symbiotic performance with the aim of obtaining high yields without N-fertilizer inputs [1, 6, 7]. Originally, Brazilian soils were free of soybean *Bradyrhizobium*[8] and commercial foreign inoculants were first used in the 20^th^ century. Searches for locally adapted strains were started immediately and still continue [1, 7, 9]. In the absence of natural biodiversity, these strain-selection programs were, and are, searches for variant genotypes of the introduced *Bradyrhizobium* strains, superior in N_2_-fixation capacity and competitiveness obtained mainly by reisolation after long periods of adaptation to local stressful environmental and soil conditions [6, 9, 10]. Two elite strains have emerged from this strategy, *B. japonicum* CPAC 15 (=SEMIA 5079) and *B. diazoefficiens* CPAC 7 (=SEMIA 5080) [9, 11]. Interestingly, either due to the adaptation to the edaphic climatic conditions, or to the selection for better symbiotic performance, several morphological, physiological and genetic differences have been reported when variant and parental strains are compared [10, 12–16]. In addition, large differences in symbiotic performance, including N_2_-fixation efficiency and competitiveness in both greenhouse controlled conditions and field experiments have also been detected between parental and variant strains [17, 18].

CPAC 15 (=SEMIA 5079) is a natural variant derived from SEMIA 566, a strain used in inoculants in the late 1960s that belongs to the same serogroup as USDA 123, which is considered as the most competitive serogroup in the United States [1, 6]. CPAC 15 was selected for higher capacity of N_2_ fixation than the parent, and it has been broadly used in commercial inoculants in Brazil since 1992 [9, 11]. This strain is the most competitive of the four commercial strains used in Brazil, and has been detected in every soil cropped with soybean [1, 6, 19, 20]. In the Midwestern United States, strains belonging to serogroup 123 have been found to occupy 60% to 80% of the nodules formed (e.g. [6, 21, 22]); even higher nodule occupancies have been reported in Brazil [20, 23].

CPAC 7 (=SEMIA 5080) is a natural variant of strain CB 1809 (=SEMIA 586, =3I1b136, =TAL 379; =USDA 136; the latter being a subculture of USDA 122 [24, 25]). CPAC 7 has been selected for higher efficiency of N_2_ fixation and higher adaptation to tropical soils than the parent [9, 11]. The strain has been used in commercial inoculants in Brazil since 1992 [11]. CPAC 7 is more efficient in fixing N_2_, but less competitive, than CPAC 15 [6, 17, 20].

Today, Brazil produces over 27 million doses of soybean inoculants per year, including exports to some South American and African countries, the great majority carrying strains CPAC 15 and CPAC 7. In this study, we report the genomes of these two strains, highlighting similarities and differences that may be related to their adaptation to tropical soils, efficiency of N_2_ fixation and competitiveness. We also compare the two genomes with those of the type strains of *B. japonicum* and *B. diazoefficiens*, and some other strains of interest.

## Results and discussion

### General characteristics of CPAC 15 and CPAC 7 genomes

**Figure 1:**
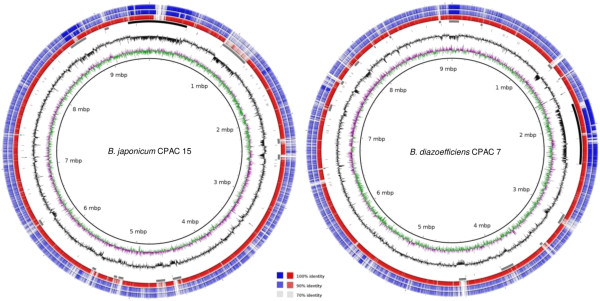
**Representation of the chromosomes of** ***B. japonicum*** **CPAC 15 and** ***B. diazoefficiens*** **CPAC 7.** Circles from innermost to outermost indicate: coordinates in Mb, GC skew, GC content, ribosomal operons, tRNA genes, genomic islands (symbiosis islands in black), BLASTN matches to *B. japonicum* USDA 6^T^ (for CPAC 15) or *B. diazoefficiens* USDA 110^T^ (for CPAC 7), BLASTN matches to CPAC 7 (for CPAC 15) or CPAC 15 (for CPAC 7), BLASTN matches to USDA 110^T^ (for CPAC 15) or USDA 6^T^ (for CPAC 7). Legend indicates BLASTN percentage identity by color.

*B. japonicum* (Bj) strain CPAC 15 has a 9,582,287-bp genome composed of one circular chromosome with two ribosomal operons and a G + C content of 63.54% (Table 1, Figure 1). Annotation predicted 4,203 ORFs with assigned function (48.6%) and 4,445 hypothetical ORFs (51.4%), according to the criteria established and described in the methods section; in total, 8,648 genes were predicted. A high-quality draft genome with 20-fold coverage and distributed in 13 contigs indicated that *B. diazoefficiens* (Bd) strain CPAC 7 has a genome size estimated at 9,085,545 bp. No plasmid replication genes were detected, indicating that CPAC 7 does not possess plasmids and that its genome is composed of a single replicon. This result is in agreement with the reported absence of plasmids in Bd strain USDA 122 [26], the parent strain of CPAC 7. In CPAC 7 there was only one ribosomal operon and the G + C content was 63.98% (Table 1, Figure 1). Annotation predicted 4,147 ORFs with assigned functions (50.2%) and 4,084 hypothetical ORFs (49.8%), with 8,231 total predicted genes. General features and statistics of both genomes are presented in Table 1.

**Table 1:** **General information about the genomes of** ***B. japonicum*** **strain CPAC 15 and** ***B. diazoefficiens*** **strain CPAC 7**

Characteristic	CPAC 15	CPAC 7
Genome coverage	20-fold	20-fold
Circular chromosome	1	1
Size (bp)	9,582,287	9,085,545
G + C content (%)	63.54	63.98
Number of contigs	1	13
Coding region (% of genome size)	82	83
Average ORF size (bp)	909	927
Number of ORFs with assigned functions	4,203	4,147
Number of hypothetical ORFs	4,445	4,084
rRNA operons	2	1
tRNA genes	51	52
Total number of genes	8,648	8,231

Despite many reports of two groups within *B. japonicum*, it was only recently that a new species, *B. diazoefficiens*, was described, which includes strains previously classified as *B. japonicum* Group Ia [3]. Average nucleotide identity (ANI) values are higher within each species than between them [3] and this is clearly observed in the identity percentage of the BLASTN matches when the genomes are compared (three outermost circles in Figure 1). Now we report that the genome of CPAC 7 is smaller than that of Bj CPAC 15 by approximately 500,000 bp. The draft genomes now available for both species (Additional file 1: Table S1) show consistently that strains belonging to *B. japonicum* have larger genomes than those of *B. diazoefficiens*.

Considering the ten organisms with the highest genomic similarities determined by the Kyoto Encyclopedia of Genes and Genomes (KEGG) system, 80.9% of the ORFs of CPAC 15 had highest similarity with Bj USDA 6^T^, 8.2% with Bd USDA 110^T^, 1.53% with *Bradyrhizobium* sp. S23321 and less than 0.4% with other organisms. In relation to CPAC 7, 81.3% of its ORFs were highest in similarity with USDA 110^T^, 5.73% with USDA 6^T^, 2.32% with S23321 and less than 0.5% for the other organisms (Additional file 1: Table S2). These results are in agreement with known taxonomic affiliations of these strains [3].

### Structural genome comparisons

**Figure 2:**
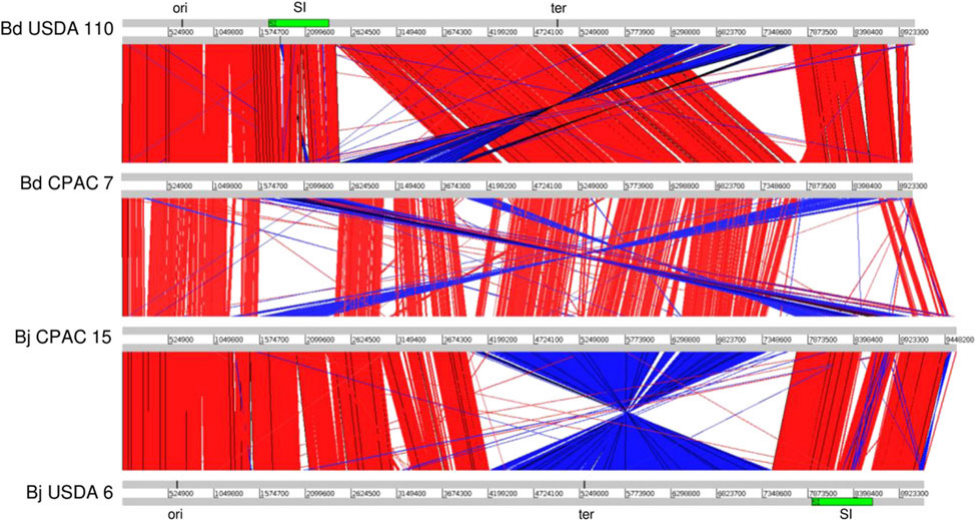
**Structural genome comparisons between the genomes of** ***B. diazoefficiens*** **strains USDA 110** ^**T**^ **and CPAC 7, and** ***B. japonicum*** **strains CPAC 15 and USDA 6** ^**T**^ . Red and blue lanes connecting genomes represent syntenic blocks in direct and reverse orientations, respectively. Positions of the replication origin (*ori*), replication terminus (*ter*) and symbiosis island (SI) are indicated.

Bj CPAC 15 and Bd CPAC 7 chromosomes were syntenic when compared with each other and with those of Bj USDA 6^T^ and Bd USDA 110^T^; however, genome rearrangements involving inversions of large genome regions, and also inversions or translocations of small regions were observed (Figure 2). As expected, synteny was higher when genomes of strains from the same species were compared; however, Bj USDA 6^T^ showed a large inversion around the chromosome terminus in comparison to Bj CPAC 15. When *B. japonicum* and *B. diazoefficiens* strains were compared, a rearrangement around the chromosome-replication origin (*ori*) that shifted the position of a region containing the symbiosis island was observed. Although the symbiosis island is located at a different replichore in each species, the orientation relative to the *ori* was preserved, suggesting that no effects on symbiotic gene expression could be attributed to this rearrangement.

In *Bradyrhizobium*, genomic plasticity involving rearrangements was previously reported when species were compared [27, 28]. Here we observed that rearrangements occur also at the strain level. In the genus *Rhizobium*, rearrangements are common but involve mainly plasmid replicons, whereas chromosomes are more stable [29, 30]. As genome plasticity may be related to adaptation [31], it is not surprising that *Bradyrhizobium* genomes also displayed rearrangements even when plasmids are relatively uncommon in this genus [26].

### Similarities and differences between protein-coding genes of *B. japonicum* and *B. diazoefficiens*

**Figure 3:**
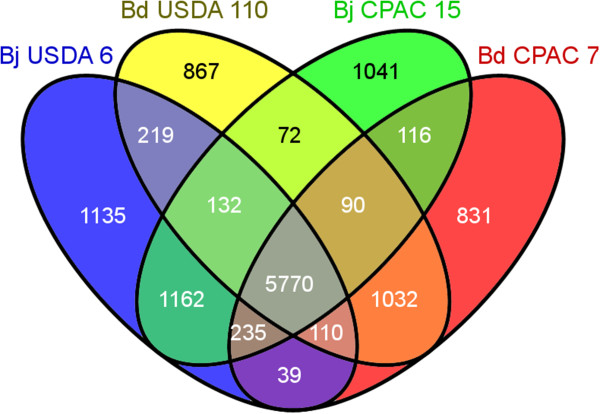
**Venn diagram depicting overlaps and differences in orthologous genes between** ***B. japonicum*** **strains USDA 6** ^**T**^ **and CPAC 15 and** ***B. diazoefficiens*** **strains USDA 110** ^**T**^ **and CPAC 7.**

Overlaps and differences in genes of Bj CPAC 15, Bj USDA 6^T^, Bd CPAC 7 and Bd USDA 110^T^ are depicted in a Venn diagram (Figure 3). The common core was composed of 5,770 genes, and included those related to basic cell survival, as ribosomal protein genes, chaperones, DNA-replication and -repair genes, hundreds of ORFs related to energy metabolism, including oxidoreductases, acyltransferases, dehydrogenases, hydrolases, cytochromes, hundreds related to carbon (C) metabolism and transporters of several classes. Other genes included in the common core had roles in nodulation and N_2_ fixation, and some were hydrogenase genes, genes related to chemotaxis and conjugal transfer, genes related to type I to VI secretion systems, interesting genes such as beta-lactamases, those linked to the metabolism of arsenate and penicillin, many transposases and, as expected, several hundreds encoding hypothetical proteins.

Bj CPAC 15 and USDA 6^T^ shared 7,299 genes, whereas Bd CPAC 7 and USDA 110^T^ shared 7,002 genes (Figure 3). In CPAC 15 and USDA 6^T^, 1,041 and 1,135 genes were exclusive to each strain, respectively. In Bj CPAC 15, 73.1% of the exclusive ORFs were classified as hypothetical (73.1%) and 5.3% as transposases; in addition, there were transporters, ORFs related to secretion systems and transcriptional regulators. In Bj USDA 6^T^, no functions could be attributed to 93.5% of its unique ORFs and 42 of them (3.7%) encoded transposases. In *B. diazoefficiens*, 831 and 867 genes were exclusive to CPAC 7 and USDA 110^T^, respectively; we must remember that these two genomes were smaller than those of *B. japonicum* (Additional file 1: Table S1). In CPAC 7, 74.6% of the exclusive ORFs were hypothetical and 5.53% encoded transposases, the remaining being represented by several classes of genes including transporters and secretion systems. In USDA 110^T^, 70.7% of the unique ORFs represented hypothetical genes, there were 88 transposase genes (7.6%), and 2.43% encoded ABC transporters. In summary, considering the whole genome of CPAC 15, 12.1% was represented by hypothetical genes exclusive to this strain. These ORFs ranged from 70 to 3,270 bp, with an average of 194 bp, and they were distributed throughout the genome. Similarly, in CPAC 7 the hypothetical exclusive genes represented 10.1% of the genome, varied in size from 70 to 2,278 bp, with an average of 190 bp, and were also broadly distributed. Altogether, this information highlights our still very poor knowledge of *Bradyrhizobium* and raises the intriguing question of what features are encoded by this high percentage of hypothetical genes.

The majority of the hypothetical ORFs in Bj CPAC 15 and Bd CPAC 7 had orthologs in at least one of the related genomes, and several had conserved domains and/or had their expression confirmed in other experiments. Some conserved domains allowed assignments in KEGG functional classes. In CPAC 15, the most representative category was that of amino acid metabolism (15.95%), followed by the xenobiotic biodegradation and metabolism (13.24%). In the case of CPAC 7, the xenobiotics category had a higher percentage (18.96%) than in CPAC 15.

Twenty-nine hypothetical proteins of CPAC 15 have been previously reported in its proteomic map [32], and now, with the complete genome, it was possible to localize them in the genome. None was found in the symbiosis island and all except one (BS07054) had orthologs in CPAC 7, USDA 110^T^ and USDA 6^T^ (Additional file 1: Table S3). The proteomic reference map of Bd CPAC 7 also presented a significant number of hypothetical sequences (unpublished) that will now be properly localized in the genome.

### Carbohydrate and amino acid metabolism

Predicted genes of Bj CPAC 15 and Bd CPAC 7 fit into 18 KEGG functional classes (Additional file 2: Figure S1). The highest percentages of ORFs were in classes of metabolism of amino acids (14.71 and 15.79% for CPAC 15 and CPAC 7, respectively) and carbohydrates (14.43 and 15.71%, for CPAC 15 and CPAC 7, respectively). The same results were obtained using the COG functional classification (data not shown). Although in RAST a smaller percentage of ORFs was assigned to functional classes, the same two categories were confirmed as the most abundant; in addition, the third class including more ORFs in both strains was that of cofactors, vitamins, prosthetic groups and pigments, followed by fatty acids, lipids and isoprenoids (Additional file 2: Figure S2).

The high number of ORFs of both strains in amino acid and carbohydrate metabolism might be related to their ability to survive in soils of low C and N content. The Brazilian Cerrados is an edaphic type of savanna occupying 207 million hectares; C content in these soils is low, ranging from 0.5 to 2.3 mg/g in the superficial layer and can decrease drastically with agricultural cultivation [33]. In a previous study, we reported that rhizobia adapted to soils of low C content may use a broader range of sources of C to survive than those in soils rich in C [34].

It is noteworthy that, in the amino acid metabolism category, CPAC 7 had a higher percentage of ORFs than did CPAC 15. It is known that a complex amino acid cycle is essential for symbiotic N_2_ fixation, involving control via amino acid exchange between the symbiotic partners, providing selective pressure for the evolution of mutualism [35, 36]. We may thus hypothesize that the higher efficiency of N_2_ fixation of CPAC 7 could be related to a larger number of genes involved in the metabolism of amino acids. It has been reported that the synthesis of branched chain amino acids (BCAAs) is switched off in bacteroids of *R. leguminosarum*, rendering them as functional auxotrophs depending on the host for their supply [37]. *In vitro* growth tests show that both Bj USDA 6^T^[3] and CPAC 15 (unpublished) use poorly the BCAAs leucine and isoleucine as sole N sources in comparison to Bd CPAC 7 and USDA 110^T^. Some genes encoding enzymes of the common and specific pathways for BCAA degradation were missing in both CPAC 15 and USDA 6^T^, therefore both strains may have to metabolize leucine and isoleucine using other less-efficient pathway. These observations may be linked to the inferior symbiotic N_2_-fixation efficiency of *B. japonicum* in comparison to *B. diazoefficiens* if leucine and isoleucine are as important in the bradyrhizobia-soybean symbioses as they are in the *R. leguminosarum*-pea (*Pisum sativum*) interaction [36].

In *Rhizobium phaseoli* CNPAF 512, mutations in genes of the arginine deiminase pathway used for arginine catabolism under low-oxygen conditions, like those occurring in nodules, diminish N_2_-fixation efficiency [38]. Interestingly, genes for the arginine deiminase pathway were found in *B. diazoefficiens* strains but not in *B. japonicum*, and, again, this may also be related to the superior N_2_-fixation ability of *B. diazoefficiens*.

### Symbiosis islands

Kaneko et al. [2, 4] proposed that the symbiosis islands of Bd USDA 110^T^ and Bj USDA 6^T^ were split in three regions, named locus A, B and C, after their insertion in the respective genomes. When data from transcriptomic studies of USDA 110^T^ are mapped into these three loci, only locus A and B showed a significant number of genes with altered expression under symbiotic conditions. Locus A is the largest region and includes the 410-kb symbiotic region of USDA 110^T^ originally described by Göttfert et al. [39]. Locus B of USDA 110^T^, 4.9 kb in size, includes only six genes, five of which are hypothetical; it is conserved in USDA 6^T^[4] and in Bj CPAC 15, but showed variability in Bd CPAC 7 (data now shown). Since locus A contains all *nod*, *nif* and *fix* genes and seems to be a *bona fide* symbiotic region we decided to focus our analysis on it.

**Figure 4:**
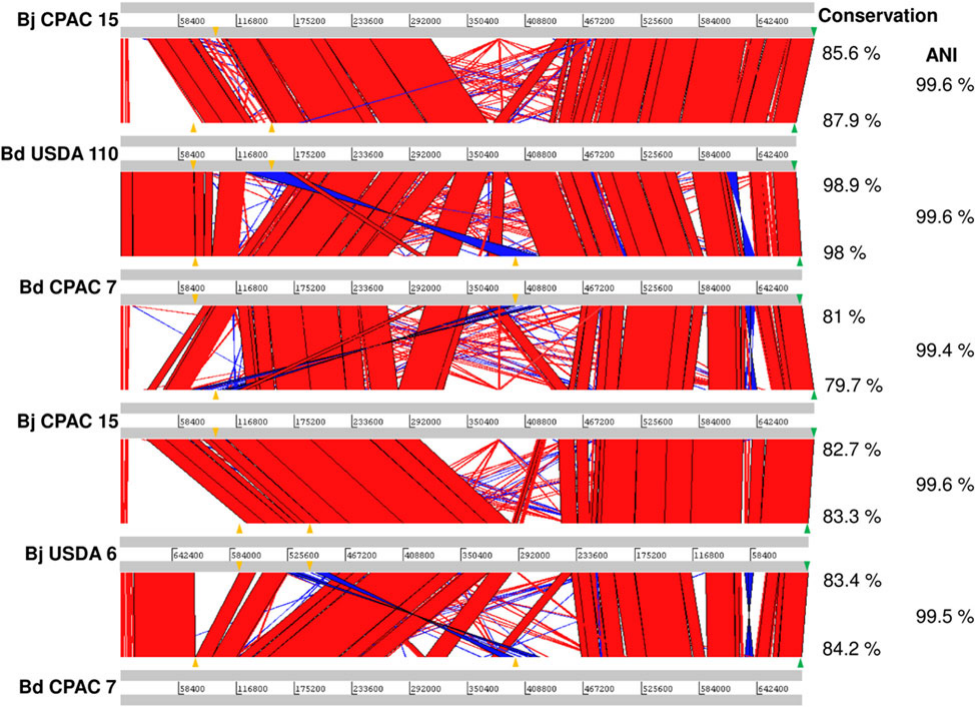
**Multiple comparison of the symbiosis islands of** ***B. diazoefficiens*** **strains USDA 110** ^**T**^ **and CPAC 7, and** ***B. japonicum*** **strains USDA 6** ^**T**^ **and CPAC 15.** Percentages of island conservation between strains are indicated at right, as well as the average nucleotide identity (ANI) values of the conserved regions. Green triangles indicate the initial tRNA-Val gene while orange triangles indicate other t-RNA genes located inside the islands.

We demarcated the beginning of the symbiosis island in a DNA-recombinase gene (BS08139 in CPAC 15 and BU02649 in CPAC 7), annotated as hypothetical proteins in the genomes of USDA 110^T^ and USDA 6^T^. The end of the symbiosis island was clearly defined as a tRNA-valine gene. Interestingly, inside the symbiosis islands there were two tRNA-methionine genes in USDA 6^T^, USDA 110^T^ and CPAC 7, and one in CPAC 15 (Table 2, Figure 4).

**Table 2:** **Properties of the symbiotic islands of** ***B. japonicum*** **and** ***B. diazoefficiens*** **strains**

	***B. japonicum***		***B. diazoefficiens***	
	USDA 6^T^	CPAC 15	USDA 110^T^	CPAC 7
Size (bp)	694,648	700,213	681,726	688,007
ORFs (total)	646	569	648	567
Hypothetical	448	247	354	294
Mobile elements	64	90	102	77
tRNA-Met	2	1	2	2

The sizes and numbers of genes identified in the symbiosis islands of the four strains are shown in Table 2. The largest size of the island of CPAC 15 seems to be related especially to a portion of the island that is unique to this strain, as shown in Figure 4. It is noteworthy that, despite a number of functional studies, a large proportion of predicted genes in the symbiosis island remains as hypothetical, representing 43% in CPAC 15 and 52% in CPAC 7. In addition, we point out that the high percentages of mobile genetic elements, of 16% for CPAC 15 and 14% for CPAC 7, in agreement with similar percentages in USDA 110^T^ and USDA 6^T^[2, 4], are likely to be implicated in genetic plasticity of the symbiosis island. The symbiosis islands of the four strains encompass all nodulation and N_2_ fixation genes required for symbioses, a probable non-functional hydrogenase operon, cytochromes for energy supply, ABC-transporters, operons for secretion systems, besides individual genes with roles not yet studied, such as a LuxR–type regulator. All these predicted genes are surrounded by several hypothetical ORFs and mobile genetic elements. The information obtained from the symbiosis island can also be related to some of our previous observations. First, reports of high rates of horizontal gene transfer from the symbiosis island of CPAC 15 to indigenous bradyrhizobia [19] might be explained by the high number of mobile genetic elements surrounding nodulation and N_2_-fixation genes, despite having a seemingly non-functional T4SS. Second, the potentially high plasticity of the symbiosis island may help to explain the extreme variability in N_2_-fixing properties among variants of the same strain, especially after adaptation to the soil [10, 14–16, 18, 19].

The symbiosis islands of the four soybean strains analyzed were not completely syntenic. There was a backbone of highly conserved regions showing ≥ 98% average nucleotide identity that was interrupted by regions unique to each strain (Figure 4). The conserved backbone regions encompassed only 80% to 88% of the islands when *B japonicum* strains were compared to each other and with the *B. diazoefficiens* strains. The most similar islands (>98% as conserved backbone) were those of *B. diazoefficiens* strains.

In the comparison of CPAC 15 and CPAC 7 it is noteworthy that the first shows higher competitiveness and the second higher efficiency of N_2_ fixation [1, 6, 9, 11, 13, 18–20]; Additional file 1: Table S4, Table S5. One first approach to explain these properties is to look for exclusive ORFs of each strain in the symbiosis islands. The Venn diagram shows that the minimum core for the symbiosis islands is composed of 241 predicted ORFs (Additional file 2: Figure S3), encompassing all nodulation, *nif* and *fix* genes, type III and IV secretion system genes, cytochromes and several ABC-transporters.

In relation to the exclusive genes of the symbiosis islands of each strain, in CPAC 15, 52% were represented by hypothetical ORFs, whereas 25% were of mobile genetic elements. In CPAC 7, these fractions were even higher, of 65% for the hypothetical and 29% for the mobile genetic element categories. For USDA 6^T^ and USDA 110^T^, the percentages of exclusive hypothetical ORFs were of 82% and 48%, while for mobile genetic elements were of 16% and 41%, respectively. We may thus conclude that the ORFs occurring exclusively in each strain and encompassing hypothetical and mobile elements ranged from 77% in CPAC 15 to 98% in USDA 6^T^. Therefore, several studies still have to be performed to understand the particularities hidden in the symbiosis islands of *Bradyrhizobium* strains.

In relation to the known genes of the symbiosis island, in both CPAC 15 and CPAC 7 we identified a main core of nodulation genes starting with *nolZY*, followed by one hypothetical protein and two ABC-transporters, *nolA*, *nodD2D1YABCSUIJ*, *nolNO*, another hypothetical ORF, *nodZ*, *fixR*, *nifA*, *fixA*, and, in CPAC 15, there were two exclusive hypothetical proteins before *fixR*; high resemblance was also found in all other *nif* and *fix* genes. Similarities of all these genes among the four strains were, in general, of 100%. A slightly lower similarity was found among *nodD2* of the four strains, and it would be interesting to develop new studies with this gene, recognized as a repressor of *nod*-gene expression [40]. The operons were thus in close agreement with the first report of the symbiosis island more than a decade ago [39].

Previously, from the genome draft of CPAC 15 [41] we discussed the role of the two-component regulatory system *nodV/nodW*, first described by Göttfert et al. [42]. In USDA 110^T^, NodV responds to an environmental stimulus, as the isoflavonoid genistein and phosphorylates the regulator NodW, which in turn may be required to positively regulate the transcription of one or several unknown genes involved in the nodulation of alternative hosts such as mung bean (*Vigna radiata*), cowpea (*Vigna unguiculata*), and siratro (*Macroptilium atropurpureum*), but not soybean. It has been suggested that NodVW recognizes different host flavonoids, or that, combined with NodD, it may increase Nod-signal synthesis [43]. *nodV/nodW* were present in the symbiosis island of CPAC 15 (BS 08331/30) and were highly similar in CPAC 7 (BU08157/58), USDA 6^T^ (BJ6T_81070/80) and USDA 110^T^ (bll1715/14). In addition, a second copy of *nodW* adjacent to the first one was present in all four strains (BS0829 in CPAC 15 and BU08159 in CPAC 7); all genes were neighbored by transposases. Interestingly, two other two-component response regulators were found in the chromosomes of all four strains, resembling *nodV/nodW*. A second copy of *nodV/nodW*, called *nwsA/nwsB*, has been previously reported [44], but the location in the genome was not clear. Here we confirm that there were two copies of *nodW* and one of *nodV* outside the symbiosis islands (the first one BS03121/22/23 in CPAC 15 and BU07026/27/28 in CPAC 7, and the second BS06847/46/45 in CPAC 15 and BU07376/77/78 in CPAC 7), also found in the chromosomes of USDA 6^T^ and USDA 110^T^. Therefore, it remains to be determined if these copies also influence host range.

Hydrogen-uptake genes were also present in the symbiosis islands of CPAC 15 and CPAC 7. Hydrogenase has been long study and associated with higher efficiency of N_2_ fixation, recycling part of the H_2_ obligatorily evolved in the reduction of atmospheric N_2_ by the nitrogenase [45, 46]. Several studies with soybean, including field experiments, have shown increases in plant growth and yield (e.g. [47, 48]). However, hydrogenases probably evolved before the N_2_-fixation process, when Earth was hydrogen enriched, such that many microorganisms—distributed through several taxa—are now able to use molecular hydrogen as an energy source [49].

Recently, some differences between USDA 110^T^ and USDA 6^T^ in their hydrogenase genes have been pointed out [4]. Now, having the genomes of CPAC 15 and CPAC 7, we can confirm the information available [4] and expand it by showing that differences in hydrogenase expression are observed at the species level. All four strains have genes encoding hydrogenase in the symbiosis island, more specifically, *hupA(=S)B(=L)CDFH, hypABFDE*. However, some of the genes are duplicated and incomplete, or are pseudogenes, such as *hupD*. The region is flanked by transposases that are also positioned between genes. Therefore, the operons might not be functional and may have gone through an evolutionary process of gene loss. Only in both *B. diazoefficiens* strains there are hydrogenase genes in other chromosomal loci, represented by almost identical genes to *hupNCUVSLCDFGHIJ*, *hypKABFCDE* and *hoxXA* with 98% to 100% gene similarity. In CPAC 7, these genes were also in a genomic island as in USDA 110^T^[4]. On the other hand, in *B. japonicum*, the only remaining gene in this region was *hupN* (BJ6T_24840 in USDA 6^T^ and BS01414 in CPAC 15). Indeed, a Hup^-^ phenotype has been reported for CPAC 15, while CPAC 7 showed a Hup^+^ phenotype [14] (Additional file 1: Table S5). In conclusion, the functional hydrogen-uptake system is located in a genomic island distinct from the symbiosis island. The effects of a functional uptake hydrogenase can thus go much further than the reported benefits in the improvement in the efficiency of N_2_ fixation.

### Other genomic islands

We identified 13 and 16 genomic islands (GIs), including the symbiosis island, in Bj CPAC 15 and Bd CPAC 7, respectively, regarded as originating from horizontal gene transfer (HGT). Several HGT events may be implicated in ecological features, such as adaptation, and thus are worthy of study [50]. The GIs of both strains were scattered throughout the genomes (Figure 1), and a list of their main features is shown in Additional file 1: Table S6. GI 9 of CPAC 15 and GI 8 of CPAC 7 corresponded to “symbiosis” locus C as defined by Kaneko et al. [4].

Almost all the GIs identified contained ORFs related to HGT functions, such as transposases, integrases and resolvases. Some included conjugal-transfer systems. Three GIs of each genome encoded restriction-modification systems that may act as mechanisms of resistance to bacteriophages or foreign DNA. Genes that may confer adaptation capabilities such as type I secretion systems and stress tolerance were also identified (Additional file 1: Table S6). It is worth mentioning that, excluding the symbiosis island, 68% and 59% of the ORFs included in GIs were hypothetical in CPAC 15 and CPAC 7, respectively. Seven and thirteen GIs in CPAC 15 and CPAC 7, respectively, were flanked by a tRNA gene. It is known that, in many cases, GIs are derived from integration events that occurred adjacent to tRNA-coding sequences [51].

CPAC 15 and CPAC 7 GIs corresponded to approximately 20% and 17% of their genomes, respectively. In comparison with CPAC 7, CPAC 15 had 3% more of its genome seemingly originating from HGT. GIs ranged from 0.009 to 0.211 Mb, and one GI of CPAC 15 (GI 1, Additional file 1: Table S6) was 0.345 Mb, larger than the others, except for the symbiosis island.

Within the symbiosis island, which is discussed later, it is noteworthy that, in the genome of CPAC 15, a region of about 75 kb contained several ORFs assigned as hypothetical (BS08270–BS08211). This region is enriched with mobile genetic elements and has two copies of putative HipA protein, in addition to another HipAB locus elsewhere. The HipAB system is associated with the stabilization of low copy-numbers plasmids in cell progeny [52], and is potentially related to successful HGT.

### Membrane transporters

Transporters—with an emphasis on ABC-transporters—are critical for bacterial adaptation, with roles in cellular physiology, including the uptake of nutrients, exclusion of cellular residues, energy generation and cellular signaling, among others [53]. Acting in several physiological roles, transporters generate functional diversity, and favor interaction with other organisms in the environment. In the case of our strains, the transporters may be of extreme importance, as the Brazilian Cerrados are very poor in nutrients, with low levels of elements critical for the N_2_-fixation process, such as phosphorus, molybdenum, and calcium [33].

ABC transporters of Bj CPAC 15 and Bd CPAC 7 represented 7.8% and 8.6% of the entire genome, respectively. The strains shared 63.81% of these genes (data not shown). CPAC 7 had more carbohydrate transporters and CPAC 15 more transporters related to uptake of opine, phosphonate and polyamines.

More genes related to phosphorus transport in CPAC 15 could represent another trait contributing to its high saprophytic capacity and competitiveness in the Cerrados soils. Also interesting was the high number of transporters of opines in CPAC 15. Opines are compounds produced by plant tumors or hairy roots induced by pathogenic *Agrobacterium* species, and represent sources of C and N for the bacteria. However, it has been reported that rhizobia—probably except for the use of nopaline in *B. japonicum*—were unable to use opine or nopaline as sources of C and N [54]. It has been suggested that utilization of these molecules could improve competiveness against other strains [55], and thus we may hypothesize that this is another trait that contributes to the superior competitiveness of CPAC 15 (Additional file 1: Table S4, Table S5). However, it is noteworthy that only in CPAC 7 we found all subunits of the transporter of manopine (BU00015 to BU00018), absent in both Bj CPAC 15 and Bj USDA 6^T^, but present in Bd USDA 110^T^ (blr3544 to blr 3547), and thus they could be specific to *B. diazoefficiens*. In addition, metabolism of manopine may also be related to tolerance of temperature stress [56], a condition typical of the Cerrados region [9, 33].

Both strains had a transporter for D-methionine (operon *metQIN*), a general L-amino acid transporter (operon *aapJQMP*) and a branched-chain amino acid transporter (operon *livKHMGF*), critical for the biological N_2_-fixation process. Monosaccharides and oligosaccharides are responsible for various processes related to bacterial growth and survival. In both genomes, ribose transporter proteins RbsB, RbsC, RbsA were found, and in addition, monosacharides may be transported by other proteins, such as GruA (present in both genomes), an analogue of the protein AraG (subunit of L-arabinose ABC transporter). In relation to oligosaccharides transporters, the sn-glycerol-3-phosphate transporter (UgpC, UgpA, UgpE) was present in both strains. A multiple sugar transporter (ChvE, GguB, GguA) was also present in both strains and, interestingly, *chvE* has been suggested as related to the higher efficiency of N_2_ fixation in variants of CPAC 15 [57]. Within the group of the ABC-2 transporters, both strains had transporters of antibiotics (*yadH*, *yadG*), lipo-oligosaccharide (*nodJ*, *nodI*), lipoproteins (*lolC*, *lolE*, *lolD*) and cell-division-related transporters (*ftsX*, *ftsE*).

In relation to the transport of other limiting nutrients in the Cerrados soils, the transport of sulphur may occur preferentially by means of the operon *cysPTWA*, closely similar in both strains, and this operon can also transport molybdate, but with a lower affinity, as shown in studies with *Eschericha coli*[58]. Other transporters of molybdenum, such as *modABC* and *mobABC* were found in both strains, as well as genes related to the transport of sulphonate and nitrate, as such as *ssuABC*.

### Surface polysaccharides

Rhizobial surface polysaccharides, such as capsular- (CPS or KPS), exo- (EPS) and lipopolysaccharides (LPS) play important roles in the symbiotic interaction with host plants [59]. Rhizobia also synthethize cell-surface cyclic beta-glucans that are involved in the symbiosis [59]. The roles of surface polysaccharides include signaling, protection against plant pathogens and nodule organogenesis [59, 60].

In many soybean bradyrhizobia, CPS and EPS have the same composition and structure, and differ only in relation to their association with the cell surface [61, 62]. The EPS of *B. japonicum* USDA 138 consists of a polymer of a pentameric repeated unit composed of (O-methyl or O-acetyl) galactose, two glucoses, O-acetyl galacturonic acid and mannose [62]. EPSs of similar sugar composition are produced by a range of soybean bradyrhizobia, including Bd strains USDA 110^T^, USDA 122 and CB 1809, and Bj USDA 123 [62, 63].

Genes related to the biosynthesis of EPS were found in CPAC 7 and CPAC 15 genomes, such as those involved in the generation of sugar precursors (*exoB*, *exoN*), assembly (*exoF*, *exoM*, *exoU*), and polymerization or transport of repeating units (*exoP*, *exoT*, *exoQ*). A conserved 11.6 kb cluster grouped genes *exoQUM, metA* and *exoPTB*. This group of genes was previously described by Becker et al. [64] in USDA 110^T^, and we noted that it is also present in USDA 6^T^. In USDA 110^T^, mutations in *exoPT* and *exoB* cause alterations in EPS composition or in the ratio of low molecular weight versus high molecular weight forms of EPS, and delayed nodulation in soybean. The EPS cluster also included genes for a glycosyltransferase and an acetyltransferase. It is worth noting that the acetyltransferase gene is shorter in USDA 110^T^ in comparison to the other strains, although the functional transferase domain seems to be complete in the predicted polypeptide. A second locus related to EPS biosynthesis has been recently identified by Quelas et al. [65] in USDA 110^T^. Mutations in *lspL* and *ugdH* of this cluster resulted in the production of EPS lacking galacturonic acid and diminished competitiveness for soybean nodulation [65]. This 14.8-kb gene cluster is conserved in CPAC 7, CPAC 15 and USDA 6^T^. Homologues to genes involved in regulation of EPS biosynthesis, such as *exoR*, *mucR* and *exoS*, were found in all four genomes, but a homologue of the *exoZ* gene required for acetylation of EPS in *S. meliloti* was present only in CPAC 15.

Species of *Bradyrhizobium* are able to produce cyclic glucans with β(1 → 6) and β(1 → 3) glycosidic linkages, containing 10 to 13 glucose residues [66]. Although cyclic β-glucans are mostly studied as secreted polysaccharides, they are predominantly located in the periplasm. These polysaccharides have been implicated in the establishment of the N_2_-fixing symbiosis in terms of suppression of plant-defense responses by their binding to receptor sites on the host-plant plasma membrane [67]. Other findings, such as the importance of cyclic β-glucans for hypo-osmotic adaptation and motility [68], and their high levels in bacteroids [69] and positive effects on isoflavonoid production in soybean [70], support their function in adaptation of rhizobia to the symbiotic lifestyle. In the four genomes analyzed, genes related to the biosynthesis of Family III glucans [71] had the same organization: *ndvBDC*. NdvB and NdvC are transferases that synthesize the cyclic glucan and a mutation in any tends to impair the symbiosis with soybean [72]. NdvD is a conserved hypothetical protein, potentially related to the transport of glucan to the periplasm [73]. A locus for Family I glucans biosynthesis composed of *mdoGH* genes was present only in the genomes of CPAC 15 and USDA 110^T^. Production of type I glucans has not been reported to date in bradyrhizobia, suggesting that expression of *mdoGH* genes require special conditions.

Molecules of LPS are major structural components of the outer membrane of Gram-negative bacteria and are typically composed of lipid A, core oligosaccharide and the O-antigen polysaccharide. Implication of LPS in several steps of the symbiotic interaction has been extensively reported [59]. Despite differences in the structure and composition, rhizobial lipid A typically contains secondary or even tertiary acyl chains. Recently, it was demonstrated that *Bradyrhizobium* strains have (ω-1)-hydroxylated very long chain fatty acids (VLCFAs), accompanied by 3-hydroxydodecanoic and 3-hydroxytetradecanoic acids; this pattern is unique to the slow-growing rhizobia [74]. Rhizobial VLCFAs are worthy of attention because of their importance to the intracellular lifestyle [75]. According to Choma & Komaniecka [4], genes related to the biosynthesis of VLCFAs in USDA 110^T^ probably correspond to bll3807–bll3811. This gene cluster was found to have the same organization in the genomes of CPAC 7, CPAC 15 and USDA 6^T^. Other *lpx* genes involved in lipid-A biosynthesis were also conserved between the analyzed strains and were located at equivalent positions in their chromosomes.

A gene cluster (*galE*-*lpcC*-*rfaF*-*rfaD*) involved in LPS’s core oligosaccharide biosynthesis has been described in *B. japonicum* 61A101C [76]. This cluster is conserved in CPAC 15 and USDA 6^T^. The O-antigen is probably the most variable portion of the LPS structure [77]. Ferreira & Hungria [8] showed that CPAC 7 and CPAC 15 have different LPS profiles in SDS-PAGE analysis, and here we show how different these profiles are (Additional file 2: Figure S4). We found strain-specific O-antigen biosynthesis gene clusters. In CPAC 7, a 30.8 kb cluster included genes for the biosynthesis of GDP-L-fucose and GDP-L-rhamnose, and *wzm*-*wzt* genes. Strain CPAC 15 had a 47.9 kb gene cluster located at the same relative chromosomal position but with distinct genes including a putative *wzx* gene. The Wzx flippase is one of the mechanisms for translocation of the O-antigen to the periplasmic space; the other mechanism requires the Wzm/Wzt ABC-type transporter. All analyzed strains belong to different serogroups (unpublished data) which is consistent with the presence of strain-specific O-antigen gene clusters in each strain genome.

### Secretion systems

#### Type I secretion system (T1SS)

Eight genes in Bj CPAC 15, Bj USDA 6^T^ and Bd USDA 110^T^, and seven in Bd CPAC 7 encoded products with glycine- and aspartate-rich repeats that are characteristic of proteins secreted by the T1SS [78]. The *B. japonicum* strains shared seven orthologous T1SS-exoprotein genes, whereas the *B. diazoefficiens* strains shared five. Two of these shared genes were present in all four genomes. Most T1SS exoproteins characterized to date belong to animal pathogens and act as toxins, proteases, lipases or adhesins which promote virulence [78]. Interestingly, the two *B. japonicum* strains and USDA 110^T^ each had one T1SS gene encoding a putative Zn-dependent metalloprotease, though the products are divergent (42-64% identity). In *Rhizobium*, T1SS exoproteins have been characterized as bacteriocins promoting competitiveness for nodulation [79], or as factors like NodO, playing a yet-unknown role in nodulation of some hosts [78]. None of the T1SS exoproteins of our strains showed similarity with known bacteriocins or NodO; nevertheless, some of these proteins bear domains related to adhesins and may be involved in competitiveness for root colonization. In *S. meliloti* 1021, the expression of a secreted peroxidase was highly induced upon oxidative stress [80]. Each *B. japonicum* strain genome encoded a putative T1SS-secreted peroxidase that might improve fitness under stressful conditions.

#### Type II secretion system (T2SS)

Genes of the T2SS molecular machinery were found only in Bd USDA 110^T^ and Bj USDA 6^T^; however, *gspC*, encoding a core component of the T2SS inner-membrane platform [81] was absent in both, indicating that strains are incapable of type II secretion. In Bj CPAC 15, a *gspO* gene remnant was located in the region corresponding to the *gsp* locus, and in Bd CPAC 7 this locus and adjacent genes seem to have been replaced by a polysaccharide-biosynthesis gene cluster. This deletion/replacement of the *gsp* locus in CPAC 15 and CPAC 7 may have been favored if the ancestor of all of the strains had a nonfunctional T2SS.

#### Type III secretion system (T3SS)

Several rhizobia, including Bd USDA 110^T^ and Bj USDA 6^T^ possess genes required for biosynthesis of T3SS in their symbiotic compartments [2, 4]. The T3SS acts as a molecular syringe to inject effector proteins into legume host cells, affecting the plant defense systems to promote colonization [82]. USDA 110^T^ mutants in the T3SS show delayed nodulation and diminished N_2_- fixation capacity with *G. max*, but are all able to normally nodulate and fix N_2_ with *V. unguiculata*[83]. The activities of T3SS effectors may have detrimental effects in some legume hosts [82] even at the cultivar level. The latter effect is exemplified by the ability of T3SS mutants of Bd USDA 122 to nodulate the soybean cultivar Hardee (*Rj*_*2*_), in which nodulation is restricted to certain bradyrhizobial strains such as USDA 122 [84]. The genomes of CPAC 15, CPAC 7, as well as USDA 6^T^ and USDA 110^T^ share a highly conserved T3SS cluster within their symbiosis island. Small differences between these clusters were observed and always involved the presence or absence of genes related to transposases and other mobile genetic elements. These differences are not expected to produce distinct phenotypes related to T3SS activity.

#### Type IV secretion system (T4SS)

Both Bj CPAC 15 and Bd CPAC 7 genomes encoded genes for T4SS. A T4SS present in CPAC 15 included all mating-pair formation (Mpf) genes required for assembly of the T4SS pilus, a set of DNA-transfer and -replication (Dtr) genes required for preparing the DNA to be transferred, and a gene for a coupling protein that delivers the DNA intermediate to the Mpf pilus [85]. This T4SS of CPAC 15 was part of GI 6 present only in this strain and may participate in conjugative transfer of this island as has been described for other T4SS [86].

Another T4SS was found encoded in the symbiosis island. However, this system seemed to be non-functional as it included only a partial set of Mpf genes, those homologous to *trbLFGI*, and a partial gene homologous to those encoding TraG-coupling proteins. These T4SS genes were found to be conserved between Bj CPAC 15 and USDA 6^T^ and Bd USDA 110^T^. The *trbLFGI* genes were not present in CPAC 7, reinforcing the possibility that this is a relic T4SS system. The symbiosis islands of all strains lacked genes for Dtr components, suggesting that the original T4SS was devoted to export effector proteins, as has been reported for the T4SS located in the *Mesorhizobium loti* R7A symbiosis island; however, we could not identify proteins similar to known T4SS effectors.

A third T4SS was identified in CPAC 15 within the GI 9. Genes for assembly of a complete Mpf pilus, for DNA processing and for a coupling protein were identified, suggesting that this system is required for conjugative transfer of this locus. GI 9 was conserved in CPAC 7 (as GI 8), USDA 110^T^ and USDA 6^T^. However, it showed some variability between strains; for example, the T4SS genes were present in USDA 110^T^ but were absent in CPAC 7 and USDA 6^T^.

#### Type V secretion system (T5SS)

Genes encoding the autotransporter T5SS subclass were found in all genomes (six in Bj 5079, four in Bj USDA 6^T^, and five in both *B. diazoefficiens* strains). Two and three of these genes in the *B. japonicum* and *B. diazoefficiens* strains, respectively, code for products possessing the pectin-lyase-type beta-helix fold at their N halves, suggesting that they may act as adhesins binding to alpha-galactose-containing polymers [87]. Putative hemagglutinin-like adhesins secreted by the two partner system T5SS subclasses were also found, two in the *B. japonicum* strains and one in the *B. diazoefficiens* strains. Three genes for probable adhesins resembling YadA of *Yersinia entercolitica* and secreted by the trimeric autotransporter T5SS subclass were identified in the *B. diazoefficiens* strains. One YadA-like gene was found in USDA 6^T^, but none in CPAC 15. Proteins secreted by the T5SS are generally regarded as virulence factors promoting host colonization by pathogenic bacteria [88], but their role, if any, in symbiotic relationships, remains to be established.

#### Type VI secretion system (T6SS)

A 14-gene locus encoding a T6SS was found in each strain genome. A between-strain comparison of these loci showed that they were syntenic and their gene products had identities larger than 91%, indicating that they are orthologous. ImpG/SciC/VasA have been suggested to act as a structural components required for T6SS functioning [89, 90]. In Bd USDA 110^T^, the gene coding for the ImpG/SciC/VasA component has undergone a mutation (generating an internal stop codon that splits it in two putative genes, bll3589 and bll3590), implying this secretion system is not active in USDA 110^T^. The T6SS molecular machinery is present in free-living pathogenic and symbiotic bacteria, and may be involved in prokaryote-eukaryote and prokaryote-prokaryote interactions [91]. The function of these secretion systems is not well understood in the context of rhizobia-legume interactions. In *R. leguminosarum*, the T6SS is required for secretion of some proteins that seem to interfere with symbiosis with pea. The wild-type strain is unable to fix N_2_, but a mutation in one of the T6SS genes allows N_2_ fixation in the host [92].

### Other possible genetic determinants of ecological traits related to colonization of soil and root

#### Type IV pili (T4P)

We found two distinct T4P gene clusters shared by the *B. diazoefficiens* and *B. japonicum* strain genomes, and two additional clusters present only in the *B. diazoefficiens* genomes. All of these T4Ps were related to the Cpa and Tad pili found in *Caulobacter crescentus* and *Aggregatibacter actinomycetemcomitans*, respectively, and thus they can be classified into subtype IVb. This T4P subtype includes those promoting host colonization of pathogenic bacteria [93], and as-yet uncharacterized T4P of other rhizobia such as *S. meliloti* 1021, *Sinorhizobium* sp. NGR234 and *Mesorhizobium huakui* MAFF303099 [94] which possess 2, 3 and 3 T4P gene clusters, respectively. In *B. diazoefficiens*, the two additional clusters may favor host colonization.

### Flagella

Bd USDA 110^T^ produces two types of flagellum, one called “thin” composed of 33-kD flagellin subunits and a “thick” one with 65-kD subunits [95]. Flagellin genes *flaC1C2C3C4* and *flaCICII*, located in different gene clusters, are required for biosynthesis of thick and thin flagella, respectively [95]. In general, the gene clusters required for biosynthesis of both types in USDA 110^T^ are conserved in Bd CPAC 7 and also in Bj CPAC 15 and USDA 6^T^; however, differences were observed in the flagellin-coding genes for the thick flagellum. Given that the flagellum encoded in the thick cluster seems to be important for competitiveness [96], it would be interesting to explore if the difference between Bj and Bd strains in correlate with competitiveness.

### Quorum sensing

Quorum sensing (QS) is a cell-cell communication mechanism that allows bacteria to regulate gene expression in response to fluctuations in cell-population density and to coordinate group behaviors [97–99]. In general, QS in Gram-negative bacteria is mediated by N-acyl-homoserine lactone (AHL)-signaling molecules, which are synthesized by LuxI protein homologs from S-adenosylmethionine (SAM) and acylated acyl-carrier proteins (acyl-ACPs). The LuxR transcriptional regulators bind to cognate HSL autoinducers to activate gene transcription of QS-target genes. QS has recently been linked to several important symbiosis features, including nodulation efficiency, symbiosome development, EPS production and N_2_ fixation [98, 99]. Approximately 22% (31 of 142) of the *B. japonicum* and *B. elkanii* strains tested produced AHLs that induced moderate to elevated levels of β-galactosidase activity with an *Agrobacterium tumefaciens* biosensor [100]. Both Bj CPAC 15 and Bd CPAC 7 strains each contain a *luxI*-type synthase, identified as BS04783 and BU02130, respectively, showing over 95% of identity to BjaI protein (blr1063) of USDA 110^T^ and with USDA 6^T^ (BJ6T_10890). Adjacent to these ORFs, there is in both genomes a *luxR*-type regulator (BS04784 and BU02129), also with high identity (>94%) to BjaR (blr1062) of USDA 110^T^ and with USDA 6^T^ (BJ6T_10880). Neither gene is in any of the genomic islands. According to Lindemann [101], BjaI and BjaR proteins of USDA 110^T^ are responsible for the synthesis of the QS signal, a branched-chain fatty acyl-HSL, isovaleryl-HSL (IV-HSL). Similar molecules may be synthesized by other strains.

In addition to the genes described above, there are isolated LuxR-type regulators in all four strains, CPAC 15 (BS08299), CPAC 7 (BU08021), USDA 110^T^ (blr1880) and USDA 6^T^ (BJ6T_79490) located in the symbiosis island, and a possible role for this gene in the regulation of the symbioses is an exciting subject for investigation.

### Iron uptake

Mechanisms that facilitate iron acquisition may confer to bacteria higher survival capacity and competitiveness [102]; even in environments not iron depleted, as is the case for the Cerrados soils [33]. Siderophores are high-affinity low-molecular-mass chelating compounds used to capture iron. In the Bj CPAC 15 and Bd CPAC 7 genomes, we found genes related to the biosynthesis of siderophores, such as citrate and cathecolate siderophore, synthesized via chorismate, in addition to genes related to iron uptake, storage and regulation.

In *B. japonicum* 61A152, citric acid can act as a siderophore [103], and Lesueur et al. [104] reported that *Bradyrhizobium* strains excrete citrate when iron-starved. CPAC 15 and CPAC 7, as well as Bd USDA 110^T^ and Bj USDA 6^T^ had three genes encoding citrate synthases. We also found in CPAC 15 and CPAC 7 genes related to the biosynthesis of cathecolate siderophores, previously isolated and characterized in *Bradyrhizobium*[105]. Nevertheless, we did not find all genes known to be in the biosynthetic pathway, e.g. isochorismate synthase and 2,3-dihydro-2,3-dihydroxybenzoate dehydrogenase were missing, but we found a chorismate synthase and isomerases that might catalyze the conversion of chorismate to isochorismate; in addition, there were several genes encoding dehydrogenase activity, which could replace the 2,3-dihydro-2,3-dihydroxybenzoate dehydrogenase [106]. We also found isochorismatases that were exclusive to CPAC 15 (BS06609, BS07416) and CPAC 7 (BU08273, BU08403), each being absent from the other three strains.

CPAC 7 possessed a gene encoding a receptor for ferric enterobactin (BU07037)—a cathecolate siderophore—which was not identified in CPAC 15. Most soil microorganisms form hydroxamate siderophore receptors [107], and the majority of receptors identified in both soybean strains were also for hydroxamate siderophores. On the other hand, CPAC 15 possessed more exclusive receptors for hydroxamate siderophores than did CPAC 7, five against one, respectively. Some rhizobial strains can utilize siderophores from other organisms by producing appropriate receptors and transporters [108], e.g., USDA 110^T^ and 61A152 are able to utilize exogenous siderophores, such as ferrichrome, rhodotorulate and pyoverdin-types [109]. These differences may permit CPAC 15 to produce and to utilize more siderophores, and to take up more iron for growth and survival, representing another feature that might help to explain its superior competitiveness compared to CPAC 7.

### Phytohormone production

Several plant hormones have been reported to positively or negatively regulate nodulation. Whereas auxins and cytokinins (CKs) are positive regulators of nodule organogenesis and development, ethylene generally blocks both infection and nodule-primordia initiation [110, 111]. In addition, we must keep in mind that rhizobia also produce phytohormones during the symbiosis that can change the phytohormone balance in the host [112].

Cytokinin is a key signaling molecule in symbiotic interactions between leguminous plants and rhizobia. Several studies have shown that CK signaling is necessary to induce cortical cell division and nodule organogenesis [110, 111, 113]. CK biosynthesis may take place by the action of the tRNA-dimethylallyltransferase (MiaA; EC 2.5.1.75), product of *miaA* gene, by a *cys*-zeatin biosynthesis route that utilizes dimethylallyl diphosphate (DMAPP) and tRNA as substrates. Alternatively, CK synthesis may involve the *ipt* gene product, an adenylate isopentenyltransferase (IPT; EC 2.5.1.27) that utilizes DMAPP and AMP, ADP or ATP as substrates in the *trans*-cytokinin pathway [114]. With *A. tumefaciens*, the gall formation is dependent on IPT activity and other CK biosynthetic proteins, which are responsible for overproduction of this hormone [114]. Like other symbionts, *Bradyrhizobium* produce CKs both *in vitro* and during the symbiosis [115–117]. In Bj CPAC 15 and Bd CPAC 7, CK production and effects on nodulation have not been studied, but both strains contain *miaA* (BS01001 and BU03527), showing 99% identity with Bd USDA 110^T^, 90% with Bj USDA 6^T^ and 76% with *Bradyrhizobium* sp strain ORS285. In all these strains, *miaA* is located adjacent to *serB* and *ilvI* genes, predicted to encode a phosphoserine phosphatase (EC 3.1.3.3) and an acetolactate synthase large subunit (EC 2.1.1.6), respectively. Therefore, CK production in CPAC 15 and CPAC 7 seems to occur via the *cys*-zeatin route, since orthologs of the *ipt* gene were not found. The importance of *miaA* has been confirmed recently in a study with a mutant of the photosynthetic *Bradyrhizobium* sp strain ORS285, revealing that tRNA degradation is the major route of CK synthesis and that the deficiency of CK production in the mutant accounts for the delay in nodulation and N_2_ fixation, as well as with smaller nodules in *Aeschynomene*[117].

Another phytohormone that positively regulates nodule formation is auxin or indole-3-acetic acid (IAA) [110]. Auxin is involved at different stages of nodule development, including the early stages of cell initiation, stimulation of early cell division, differentiation of nodules and in the systemic regulation of nodule numbers [112]. Because many rhizobia are capable of producing IAA via different pathways, it is also assumed that bacterially produced auxin can alter the balance inside the plant. In addition, rhizobia can indirectly influence auxin homeostasis by interfering with plant-auxin transport [112]. *Bradyrhizobium* strains synthesize auxin [14, 116, 118–120], and effects of IAA produced by *Bradyrhizobium* on the symbiosis have been reported. Nodules of soybean inoculated with a *B. japonicum* IAA-overproducing mutant have bacteroids with higher IAA content [121, 122], and positive and negative effects of this increased level on nodulation have been reported [121, 123], indicating that IAA level in nodules are tightly regulated, but that the role of IAA in the symbiosis is not clear yet. *In vitro* CPAC 15 produces more IAA than CPAC 7 (Additional file 1: Table S5).

In bacteria, at least five pathways have been described for the synthesis of IAA [124–126] starting from tryptophan. The indole-3-acetamide (IAM) route is the most studied, particularly in plant pathogens such as *A. tumefaciens* and *Pseudomonas savastanoi*[124, 126, 127]. In this pathway, Trp is first converted into IAM by a Trp 2-monooxygenase (TMO), and then to IAA by IAM-hydrolase (IaaH). In *A. tumefaciens* and *Pseudomonas savastanoi*, the genes encoding TMO (*tms-1* or *iaaM*) and IaaH proteins (*tms-2* or *iaaH*) constitute an operon and are associated with pathogen virulence and gall formation in plants [124, 126]. The existence of the IAM route in *Bradyrhizobium* sp. strains has been considered based on the detection of IAM *in vitro* and also by the determination of IAM-hydrolase activity and sequencing of the *bam* gene, an ortholog of the *iaaH* gene [118–120]. Three ORFs in the CPAC 15 (BS05569, BS01233, BS02084) and two in the CPAC 7 (BU07366, BU07366) genomes showed high similarity to *bam* and *iaaH* genes. BS05569 and BU07366 are clustered with ORFs involved in cysteine and methionine metabolism (*metA* and *metY*) and with ORFs encoding a polysaccharide deacetylase and a 1-aminocyclopropane-1-carboxylate deaminase (*acdS*) related to ethylene biosynthesis. BS01233 and BU07366 are clustered with a glutathione peroxidase, whereas BS02084, exclusive to CPAC 15, is positioned together with an indolepyruvate ferredoxin oxidoreductase (IOR, EC 1.2.7.8). The other enzyme of the IAM route, the Trp 2-monooxygenase (TMO), was not found in the vicinity of any ORF encoding IAM-hydrolase protein and blast searches with *tms-1* and *iaaH* orthologs against CPAC 15 and CPAC 7 genomes were not successful. Previously, attempts to amplify the TMO gene and to determine TMO activity of *Bradyrhizobium* strains failed [120]. In addition, the genome analysis of CPAC 15 and CPAC 7 supports the proposal that, in *Bradyrhizobium*, the synthesis of IAM is independent of TMO activity and related to action of nitrile hydratase (Nhase; EC 4.2.1.84), which converts indol-3-acetonitrile (IAN) into IAM [120].

The genes for cobalt-containing nitrile hydratase (Nhase) α and β subunits (*nthA* and *nthB*) were located in the genomes of CPAC 15 (BS03457 and BS03458) and CPAC 7 (BU04142 and BU04143) downstream of an ORF encoding a Nhase activator protein (BS 03459 or BU04144, respectively), which is required for full activity of their respective NHase. Another enzyme involved in IAA biosynthesis via indol-3-acetonitrile (IAN) is nitrilase (Nit; EC 3.5.5.1), which converts IAN directly into IAA. CPAC 7 contained a nitrilase gene (BU00160) with high similarity to that of USDA 110^T^ (blr3397), reported to be an aliphatic nitrilase with high affinity for hydrocinnamonitrile and with low activity towards indol-3-acetonitrile [128]. In CPAC 15, three genes showing similarity to nitrilase (BS06879, BS00934, BS02760) were found; interestingly, BS02760 is adjacent to an ORF predicted to encode an acetaldoxime dehydratase, which catalyzes IAN production via intermediate indole-3-acetaldoxime (IAOx).

It is noteworthy that Boddey & Hungria [14] reported that the capacity for synthesis of IAA *in vitro* by CPAC 15 is greater (31.5 μM) in comparison to CPAC 7, USDA 110^T^ and USDA 123 (13.12, 6.85 and 4.88 μM, respectively), what could be explained by the higher number of ORFs detected in CPAC 15. This feature should be explored more thoroughly, as the use of *Bradyrhizobium* as plant-growth promoting rhizobacteria (PGPR) of non-legumes has been suggested [129]. The delivery of phytohormones straight to plants is suggested by the findings that *Bradyrhizobium* is found as an endophyte of non-legumes, such as wild rice (*Oryza breviligulata*) in Senegal and Guinea [130] and sugarcane (*Saccharum* spp.) in Brazil [131]. Therefore, the study of elite *Bradyrhizobium* strains, such as CPAC 15, may have important implications beyond the symbiosis with soybean. Contributions of *Bradyrhizobium* to the N-nutrition of non-legumes is also worth investigating given recent findings of bradyrhizobial *nifH* transcripts inside sugarcane [132].

A transcriptomic study with USDA 110^T^ incubated with 1 mM IAA resulted in 1,323 genes differentially expressed, most related to responses to heat, cold, oxidative, osmotic and desiccation stresses and in EPS biosynthesis [133]. Therefore, again, IAA may play several roles.

### Secondary metabolism

Although secondary metabolites are not essential for growth and reproduction of organisms, they represent natural molecules of adaptation [134], playing several roles, including development of the symbioses, responses to predators, as well as antibiotics and effectors in ecological competitiveness [134–136]. Several secondary metabolic pathways were identified in both Bj CPAC 15 and Bd CPAC 7, with the largest numbers being related to the biosynthesis of butirosin and neomycin (aminoglycoside family). Although several ORFs related to the isoquinoline alkaloid pathway were found, the majority was repeated or encoded the same protein. These genes are responsible for subpathways for galanthamine, colchicine and kreysigine synthesis. In both strains, within the pathway for stilbenoid, diarylheptanoid and gingerol biosynthesis, there was a subpathway for the production of 6-gingerol and curcumin, two compounds showing antitumor activity [137]. Other rhizobial molecules have been shown to possess antitumor activity [138].

ORFs related to the hexosyltransferase family (EC 2.4.1.-), the “transferring groups other than amino-acyl groups” family (EC 2.3.1.-) and the methyltransferase family (EC 2.1.1.-) were present in high number in the anthocyanin biosynthesis pathway in both strains. These compounds have antibiotic, antiviral and antifungal activities [139] and may contribute to strain survival in soil.

Both strains have genes related to the conversion of DIBOA-glucoside in TRIBOA-glucoside and DIMBOA-glucoside, and most ORFs for benzoxazinoid pathway show that this subpathway may be functional. Usually benzoxazinones are released by plants and have antifungal and antibacterial activities [140] and being metabolized by strains CPAC 15 and CPAC 7 may represent an advantage in terms of survival.

In relation to the biosynthesis of puromycin, novobiocin and streptomycin, we identified genes in both genomes, but the biosynthesis pathways were incomplete in both strains. In the puromycin pathway (*pur* cluster), we found *pur10*, *pur4*, and *pur5* in both strains, and a putative *pur7* in CPAC 15, suggesting that it is capable of metabolizing ATP into 3'-amino-3'-deoxy-AMP, which can then be used in other secondary biosynthetic processes. Similarly for streptomycin-biosynthesis, *algC* (phosphomannomutase/phosphoglucomutase) and *glk* (glucokinase) were present, capable of converting D-glucose-1-phosphate and D-glucose into D-glucose-6-phosphate, respectively, which can then be used for other purposes including biosynthesis of butirosin and neomycin.

The high number of ORFs related to secondary metabolites in both strains might be related to the ecology of these bacteria, in particular to the adaptation to the Cerrados soils. In addition, in almost all categories of secondary metabolites CPAC 15 had higher numbers of ORFs than CPAC 7 (Additional file 2: Figure S5), possibly related to the superior saprophytic capacity and competitiveness of the former.

In a second approach, both genomes were analyzed to find genes or gene clusters that encode proteins involved in secondary metabolite biosynthesis using the antiSMASH software [141] (Additional file 1: Table S7). Both CPAC 15 and CPAC 7 genomes encoded a two-gene cluster for bacteriocin synthesis. Bacteriocins are important in competitiveness of rhizobial strains. It has been reported that bacteriocin-producing strains have higher capacity for competitiveness and occupancy of nodules [142]. In addition, both strains share gene clusters responsible for the production of terpenes, chains of isoprene units, and peptide secondary metabolites. Only CPAC 15 encoded a type I polyketide synthase, a multi-domain enzyme that may be responsible for the synthesis of a yet unknown compound.

## Conclusion

The soybean-*Bradyrhizobium* symbiosis is considered as one of the most efficient in fixing N_2_ and probably the highest in global economic importance. Gaining a better understanding of the microsymbiont is thus important as it can open new frontiers for the improvement of the symbiosis not only with soybean, but also with other legumes. Here we described the genomes of two inoculant elite strains, Bj CPAC 15 and Bd CPAC 7—applied yearly to millions of hectares in Brazil, other countries of South America and, more recently, in African countries—as well as the genome comparison with *B. japonicum* and *B. diazoefficiens* type strains. Most of the genomes were syntenic. However, genome rearrangements were observed both between and within species, conferring high genomic plasticity, which may generate environmental adaptability and contribute to differences in symbiotic performance. Our still-poor knowledge of these important bacteria was emphasized with the observation that it was not possible to attribute any known function to about 50% of the predicted genes, including genes of the symbiosis island. The high proportion of ORFs related to the metabolism of amino acids and carbohydrates of CPAC 7 and CPAC 15 might be related to the broad adaptability of both strains to tropical soils with low N and C contents. CPAC 15, outstanding in saprophytic capacity and competitiveness had more genes in all categories of secondary metabolites, transporters of nutrients, iron-acquisition and IAA metabolism. In CPAC 7, characterized by higher efficiency of N_2_ fixation, more ORFs were related to the metabolism of amino acids, particularly in the metabolism of leucine, isoleucine and arginine; and a functional hydrogenase operon. Other differences between the species were detected, including size of the genomes, number of ribosomal operons, genes related to hydrogenase activity, LPS and type IV pili. Interesting genes were predicted in all strains, including those related to type I, III, IV, V and VI secretion systems, EPS, quorum sensing and genes related to the synthesis of phytohormones, among others. Although the backbone of the symbiotic island was conserved among the strains, the conserved region was interrupted by regions unique to each strain and many hypothetical ORFs and mobile elements that may contribute to differences in symbiotic performance.

## Methods

### Background information about CPAC 15 and CPAC 7

*B. japonicum* (Bj) CPAC 15 (=SEMIA 5079, = DF 24, =CNPSo 7) shows high saprophytic capacity (i.e. capable of surviving for long periods in soils even under nutrient-limiting and environmental stressing conditions) and high competitiveness in comparison with other soybean *Bradyrhizobium* strains, whereas *B. diazoefficiens* (Bd) CPAC 7 (=SEMIA 5080, =CNPSo 6) has higher efficiency of N_2_ fixation but lower competitiveness and saprophytic capacity than CPAC 15 [1, 6, 9, 11, 13, 18–20]. Both strains are well adapted to tropical environmental stressful conditions, including high temperature and low soil moisture and fertility, with an emphasis on soil acidity and low phosphorus content. Some morphological, physiological, symbiotic and genetic differences previously reported between the two strains are summarized in Additional file 1: Table S5.

### Bacterial strains and growth conditions

Bj strain CPAC 15 and Bd strain CPAC 7 were obtained from the “Diazotrophic and Plant Growth Promoting Bacteria Culture Collection of Embrapa Soja” (WFCC Collection # 1213, WDCC Collection # 1054) located in Londrina, Paraná, Brazil. CPAC refers to Embrapa Cerrados, Planaltina, Federal District, Brazil. Bacterial growth conditions and DNA extraction were performed as described before [41].

### Sequencing, assembly and gap closure

Library generation and genome sequencing of both strains were performed at “Darcy Fontoura de Almeida” Computational Genomics Unity (UGCDFA) of the National Laboratory of Scientific Computation (LNCC) (Petrópolis, Rio de Janeiro, Brazil). The genomes were sequenced using a whole-genome shotgun strategy, with a combination of Roche 454 GS-FLX shotgun and 3 kb-insert paired-end libraries. Libraries were prepared following GS FLX Titanium series protocols. 454 sequence reads were assembled using both Newbler 2.6 (454 Life Sciences, Roche Diagnostics Corporation, Branford, CT) and Celera (WGS, version 7.0) assemblers. The two assemblies were aligned to each other with Cross Match (Phred/Phrap/Consed package) since both yielded results that can be complementary and effective to close gaps. Sanger sequences previously obtained for strain CPAC 15 at Embrapa Soja (Brazil) [41] were also joined to the pyrosequence data. For closing gaps, a primer walking strategy was used, PCR products were Sanger sequenced at Embrapa Soja. All the consensus sequences of each contig and the sequence that closed each gap were aligned and joined using Consed (version 20.0). Information for scaffolding and gap closure was also obtained by mapping each assembly against the reference genomes of *B. diazoefficiens* USDA 110^T^ (BA000040.2) and *B. japonicum* USDA 6^T^ (AP012206.1).

### Annotation

Annotation and analysis of the sequences were carried out using the System for Automated Bacterial Integrated Annotation (SABIA) [143]. An automatic functional annotation was performed using the KEGG database according to the following criteria: *i)* ORFs with a BLASTP hit on KEGG database with a minimum 50% similarity, 60% query coverage and 80% subject coverage were assigned “valid”. The first three hits were analyzed and the product was imported from KEGG Orthology (KO) if there was one associated with the hit, or from KEGG GENES definition if no KO was associated with the first three hits; *ii)* ORFs that had (1) no BlastP hits found on the NCBI-nr, KEGG, UniProtKB/Swiss-Prot, TCDB and Interpro databases, or (2) the first three BlastP hits product on KEGG containing the keyword hypothetical were assigned “hypothetical”. A manual annotation by comparison with the UniProt/Swiss-Prot, KEGG, NCBI-nr and InterPro databases was performed for ORFs that did not fit the above criteria. In addition, annotation was also performed using the Rapid Annotation using Subsystem Technology (RAST) server [144]. Data for CPAC 15 and CPAC 7 were submitted to the GenBank database and were assigned Bioprojects numbers PRJNA20963 and PRJNA47329, respectively. Accession numbers for the genomes at the GenBank are CPAC 7 (ADOU00000000) and CPAC 15 (CP007569).

### Comparative genomics and other bioinformatics analyses

Genome alignments of CPAC 15, CPAC 7, USDA 110^T^ and USDA 6^T^ were performed with Mummer [145] and Mauve [146]. The genomes were also analyzed by the Bidirectional Best-Hits (BBH) clustering method [147] that compares the genome of each strain against each of the other genomes, using the BLAST program [148] to identify pairs of corresponding genes (clusters) and to recognize the best “hit” in other genomes. The parameters applied were 60% coverage, 60% of similarity and an E-value of <10^-5^_._ Venn diagrams were built with GeneVenn and VENNY [149, 150] to compare the list of shared and unique genes.

For transporters, a more detailed analyses was performed with the Transporter Classification Database—TCDB database [151]. Another database used was PFAM [152] to find domains related to the ABC 1, 2 and 3 transporters. Once the information was found, a search in the *in silico* proteome of CPAC 15 and CPAC 7 was performed with HMMER [153] to search for similarities in the protein sequences using an E-value <10^-4^.

Genomic islands were predicted using IslandViewer [154]. Search for T4SS effector proteins were performed using the SecReT4 web server [155]. Other software used for specific analyses were cited in the text.

### Availability of supporting data

Supporting data are included as additional files. In addition, detailed information about the genomes are available at the homepages:

http://ligeirinha.lncc.br/bj5079-final-bin/general_info.cgi for *B. japonicum* CPAC 15; http://ligeirinha.lncc.br/bj5080-final-bin/general_info.cgi for *B. diazoefficiens* CPAC 7.

## Electronic supplementary material

Additional file 1: **Supplementary tables.** (DOCX 80 KB)

Additional file 2: **Supplementary figures.** (DOCX 1 MB)
