# Supplementary material for: Comparative genomics of Bradyrhizobium japonicum CPAC 15 and Bradyrhizobium diazoefficiens CPAC 7: elite model strains for understanding symbiotic performance with soybean
Source: BMC Genomics. 2014 Jun 3;15(1):420. doi: 10.1186/1471-2164-15-420 (PMC4070871; doi:10.1186/1471-2164-15-420)
Supplement: Supplementary file 1 — Additional file 1: Supplementary tables. (DOCX 80 KB) [file 12864_2014_6142_MOESM1_ESM.docx]

## Table S1 Genome sizes of *B. japonicum* and *B. diazoefficiens* strains

| *Bradyrhizobium japonicum* | | |  | *Bradyrhizobium diazoefficiens* | | |
| --- | --- | --- | --- | --- | --- | --- |
| Strain | Size (bp) | Reference |  | Strain | Size (bp) | Reference |
| USDA 6^T^ | 9,207,384 | Kaneko et al. (2011) |  | USDA 110^T^ | 9,105,828 | Kaneko et al. (2002) |
| USDA 123 | 10,457,665 | DOE-JGI |  | USDA 122 | 8,976,980 | DOE-JGI |
| USDA 38 | 9,608,975 | DOE-JGI |  | CPAC 7 | 9,085,545 | This study |
| CPAC 15 | 9,582,287 | This study |  | CCBAU41267 | 9,140,000^*^ | Tian et. al. (2012) |
| CCBAU15354 | 10,60,00^*^ | Tian et. al. (2012) |  |  |  |  |
| CCBAU25435 | 9,480,00^*^ | Tian et. al. (2012) |  |  |  |  |
| CCBAU15618 | 9,860,00^*^ | Tian et. al. (2012) |  |  |  |  |
| CCBAU15517 | 9,940,00^*^ | Tian et. al. (2012) |  |  |  |  |
| CCBAU83623 | 10,120,00^*^ | Tian et. al. (2012) |  |  |  |  |

## ^*^ Size estimated based on scaffold information.

## Kaneko T, Maita H, Hirakawa H, Uchiike N, Minamisawa K, Watanabe A, Sato S: Complete genome sequence of the soybean symbiont *Bradyrhizobium japonicum* strain USDA6^T^. *Genes* 2011, 2(4):763-787.

## Kaneko T, Nakamura Y, Sato S, Minamisawa K, Uchiumi T, Sasamoto S, Watanabe A, Idesawa K, Iriguchi M, Kawashima K *et al*: Complete genomic sequence of nitrogen-fixing symbiotic bacterium *Bradyrhizobium japonicum* USDA110. *DNA Res* 2002, 9(6):189-197.

## Tian CF, Zhou YJ, Zhang YM, Li QQ, Zhang YZ, Li DF, Wang S, Wang J, Gilbert LB, Li YR *et al*: Comparative genomics of rhizobia nodulating soybean suggests extensive recruitment of lineage-specific genes in adaptations. *Proc Natl Acad Sci USA* 2012, 109(22):8629-8634.

## Table S2 List of the ten organisms with the highest similarities with *B. japonicum* CPAC 15 (database of 8,648 ORFs) and *B. diazoefficiens* CPAC 7 (database of 8,236 ORFs). Based on KEGG functional classification

|  | CPAC 15 | | CPAC 7 | |
| --- | --- | --- | --- | --- |
|  | Organism | Total ORFs (%) | Organism | Total ORFs (%) |
| 1 | [*Bradyrhizobium japonicum* USDA 6](http://ligeirinha.lncc.br/bj5079-bin/annotation/listOrfsbyOrg.cgi?code=bju)^T^ | 7001 (80.9 %) | [*Bradyrhizobium diazoefficiens* USDA 110](http://ligeirinha.lncc.br/bj5080-bin/annotation/listOrfsbyOrg.cgi?code=bja)^T^ | 6697 (81.3 %) |
| 2 | [*Bradyrhizobium diazoefficiens* USDA 110](http://ligeirinha.lncc.br/bj5079-bin/annotation/listOrfsbyOrg.cgi?code=bja)^T^ | 712 (8.2 %) | [*Bradyrhizobium japonicum* USDA 6](http://ligeirinha.lncc.br/bj5080-bin/annotation/listOrfsbyOrg.cgi?code=bju)^T^ | 473 (5.7 %) |
| 3 | [*Bradyrhizobium* sp. S23321](http://ligeirinha.lncc.br/bj5079-bin/annotation/listOrfsbyOrg.cgi?code=brs) | 132 (1.5 %) | [*Bradyrhizobium* sp. S23321](http://ligeirinha.lncc.br/bj5080-bin/annotation/listOrfsbyOrg.cgi?code=brs) | 191 (2.3 %) |
| 4 | [*Bradyrhizobium* sp. BTAi1](http://ligeirinha.lncc.br/bj5079-bin/annotation/listOrfsbyOrg.cgi?code=bbt) | 33 (0.38 %) | [*Nitrobacter hamburgensis*](http://ligeirinha.lncc.br/bj5080-bin/annotation/listOrfsbyOrg.cgi?code=nha) | 35 (0.42 %) |
| 5 | [*Nitrobacter hamburgensis*](http://ligeirinha.lncc.br/bj5079-bin/annotation/listOrfsbyOrg.cgi?code=nha) | 25 (0.29 %) | [*Bradyrhizobium* sp. BTAi1](http://ligeirinha.lncc.br/bj5080-bin/annotation/listOrfsbyOrg.cgi?code=bbt) | 30 (0.36 %) |
| 6 | [*Bradyrhizobium* sp. ORS 278](http://ligeirinha.lncc.br/bj5079-bin/annotation/listOrfsbyOrg.cgi?code=bra) | 22 (0.253 %) | [*Bradyrhizobium* sp. ORS 278](http://ligeirinha.lncc.br/bj5080-bin/annotation/listOrfsbyOrg.cgi?code=bra) | 24 (0.292 %) |
| 7 | [*Rhodopseudomonas palustris* DX-1](http://ligeirinha.lncc.br/bj5079-bin/annotation/listOrfsbyOrg.cgi?code=rpx) | 13 (0.15 %) | [*Rhodopseudomonas palustris* TIE-1](http://ligeirinha.lncc.br/bj5080-bin/annotation/listOrfsbyOrg.cgi?code=rpt) | 7 (0.08 %) |
| 8 | [*Rhodopseudomonas palustris* BisA53](http://ligeirinha.lncc.br/bj5079-bin/annotation/listOrfsbyOrg.cgi?code=rpe) | 11 (0.132 %) | [*Variovorax paradoxus* S110](http://ligeirinha.lncc.br/bj5080-bin/annotation/listOrfsbyOrg.cgi?code=vap) | 7 (0.08 %) |
| 9 | [*Burkholderia phymatum*](http://ligeirinha.lncc.br/bj5079-bin/annotation/listOrfsbyOrg.cgi?code=bph) | 9 (0.10 %) | [*Parvibaculum lavamentivorans*](http://ligeirinha.lncc.br/bj5080-bin/annotation/listOrfsbyOrg.cgi?code=pla) | 6 (0.07 %) |
| 10 | [*Methylobacterium* sp. 4-46](http://ligeirinha.lncc.br/bj5079-bin/annotation/listOrfsbyOrg.cgi?code=met) | 9 (0.10 %) | [*Mesorhizobium ciceri*](http://ligeirinha.lncc.br/bj5080-bin/annotation/listOrfsbyOrg.cgi?code=mci) | 5 (0.06 %) |

**Table S3 ORFs assigned as hypothetical proteins in *B. japonicum* CPAC 15 genome and previously identified in the proteomic map of this strain (Batista et al., 2010)**

| **ORFs in the genome** | **Protein identified** |
| --- | --- |
| BS01922 | [Bll7437](http://onlinelibrary.wiley.com/doi/10.1002/pmic.201000092/full#fn8) |
| BS06526 | [Blr3161](http://onlinelibrary.wiley.com/doi/10.1002/pmic.201000092/full#fn8) |
| BS03277 | [Bll4589](http://onlinelibrary.wiley.com/doi/10.1002/pmic.201000092/full#fn8) |
| BS04123 | [Blr3921](http://onlinelibrary.wiley.com/doi/10.1002/pmic.201000092/full#fn8) |
| BS02027 | [Blr7528](http://onlinelibrary.wiley.com/doi/10.1002/pmic.201000092/full#fn8) |
| BS03669 | [Bll4367](http://onlinelibrary.wiley.com/doi/10.1002/pmic.201000092/full#fn8) |
| BS04990 | [Blr0845](http://onlinelibrary.wiley.com/doi/10.1002/pmic.201000092/full#fn8) |
| BS07143 | [Blr2510](http://onlinelibrary.wiley.com/doi/10.1002/pmic.201000092/full#fn8) |
| BS03934 | [Blr4087](http://onlinelibrary.wiley.com/doi/10.1002/pmic.201000092/full#fn8) |
| BS05244 | [Blr0617](http://onlinelibrary.wiley.com/doi/10.1002/pmic.201000092/full#fn8) |
| BS07054 | [Blr2474](http://onlinelibrary.wiley.com/doi/10.1002/pmic.201000092/full#fn8) |
| BS06230 | [Blr2865](http://onlinelibrary.wiley.com/doi/10.1002/pmic.201000092/full#fn8) |
| BS07293 | [Blr2372](http://onlinelibrary.wiley.com/doi/10.1002/pmic.201000092/full#fn8) |
| BS03297 | [Blr4568](http://onlinelibrary.wiley.com/doi/10.1002/pmic.201000092/full#fn8) |
| BS00361 | [Blr5933](http://onlinelibrary.wiley.com/doi/10.1002/pmic.201000092/full#fn8) |
| BU01352 | [Blr2267](http://onlinelibrary.wiley.com/doi/10.1002/pmic.201000092/full#fn8) |
| BS07168 | [Blr2487](http://onlinelibrary.wiley.com/doi/10.1002/pmic.201000092/full#fn8) |
| BS05583 | [Blr0227](http://onlinelibrary.wiley.com/doi/10.1002/pmic.201000092/full#fn8) |
| BS04270 | [Bll3794](http://onlinelibrary.wiley.com/doi/10.1002/pmic.201000092/full#fn8) |
| BS07158 | [Bll2497](http://onlinelibrary.wiley.com/doi/10.1002/pmic.201000092/full#fn8) |
| BS01146 | [Bll6649](http://onlinelibrary.wiley.com/doi/10.1002/pmic.201000092/full#fn8) |
| BS03196 | [Bll4707](http://onlinelibrary.wiley.com/doi/10.1002/pmic.201000092/full#fn8) |
| BS07759 | [Bll1169](http://onlinelibrary.wiley.com/doi/10.1002/pmic.201000092/full#fn8) |
| BS02485 | [Bll5551](http://onlinelibrary.wiley.com/doi/10.1002/pmic.201000092/full#fn8) |
| BS07759 | [Bll1169](http://onlinelibrary.wiley.com/doi/10.1002/pmic.201000092/full#fn8) |
| BS01852 | [Bll7369](http://onlinelibrary.wiley.com/doi/10.1002/pmic.201000092/full#fn8) |
| BS04577 | [Bll7953](http://onlinelibrary.wiley.com/doi/10.1002/pmic.201000092/full#fn8) |

## Batista JS, Torres AR, Hungria M: Towards a two-dimensional proteomic reference map of *Bradyrhizobium japonicum* CPAC 15: spotlighting "hypothetical proteins". *Proteomics* 2010, 10(17):3176-3189.

## Table S4 Comparison of symbiotic performance of *B. japonicum* CPAC 15 and *B. diazoefficiens* CPAC 7 and in a field experiment performed in an oxisol in Brazil cropped for the first time with soybean in originally free of soybean bradyrhizobia. Inoculation was performed by adding 1.2 million cells of the inoculant strain per seed.

| Treatment | 40 days after emergence | | | | | |  | Final harvest |
| --- | --- | --- | --- | --- | --- | --- | --- | --- |
|  | Nodulation  ________________________________ | | | | Plant Biomass  _____________ | |  | Grain  _____ |
|  | Number | Dry weight | Nodule occupancy by inoculated strain (%) | | Total N in shoots | Nodule efficiency |  | Yield |
|  | (n° pl^-1^) | (mg pl^-1^) | 1st year | 2nd year (survival) | (mg N pl^-1^) | (mg N mg nodule^-1^) |  | (kg ha^-1^)_ |
| Non-inoculated | 9 b* | 16.2 b | - | - | 18 b | 1.11 c |  | 886 c |
| Non-inoculated + 200 kg N ha-1 | 4 b | 9.3 b | - | - | 135 a | - |  | 3021 b |
| *B.* *japoncium* CPAC 15 | 48 a | 79 a | 92 | 90 | 151 a | 1.91 b |  | 3328 ab |
| *B. diazoefficiens* CPAC 7 | 33 a | 62 a | 80 | 48 | 223 a | 3.60 a |  | 3545 a |

## The data represent the means of six replicates and when followed by the same letter, within the column, do not show statistical difference (Tukey, *p*<0.05).

**Table S5 Morphological, physiological and genetic differences between *B. japonicum* CPAC 15 and *B. diazoefficiens* CPAC 7 reported in previous studies by our group**

| **Characteristic** | **CPAC 15** | **CPAC 7** | **Reference** |
| --- | --- | --- | --- |
| Antibiotic resistance |  |  |  |
| kan15 | y | y | [[1](#_ENREF_1)] |
| rif25 | y | y | [[1](#_ENREF_1)] |
| spe10 | n | n | [[1](#_ENREF_1)] |
| str40 | y | n | [[1](#_ENREF_1)] |
| str80 | y | n | [[1](#_ENREF_1)] |
| str100 | y | n | [[1](#_ENREF_1)] |
| car500 | y | n | [[1](#_ENREF_1)] |
| chl500 | n | n | [[1](#_ENREF_1)] |
| ery250 | y | n | [[1](#_ENREF_1)] |
| nal50 | y | y | [[1](#_ENREF_1)] |
| rif500 | y | y | [[1](#_ENREF_1)] |
| tet100 | y | y | [[1](#_ENREF_1)] |
| Physiological and symbiotic properties | | | |
| IAA | 31.46 | 24.88 | [[1](#_ENREF_1)] |
| Hup | Hup- | Hup+ | [[1](#_ENREF_1)] |
| Tolerance to 0.3M NaCl | n | n | [[2](#_ENREF_2)] |
| Growth at 4⁰C | n | n | [[2](#_ENREF_2)] |
| Nodulation cv. Hill (Rj4) | n | y | [[1](#_ENREF_1)] |
| Rhizobiotoxin symptons in alfalfa roots | y | n | [[1](#_ENREF_1)] |
| Chlorose symptons (rhizobiotoxin) on leaves of cv. BR 16 and Lee | y | n | [[1](#_ENREF_1)] |
| Nodulation | higher | lower | [[3](#_ENREF_3)] |
| N_2_ fixation efficiency | lower | higher | [[2](#_ENREF_2), [3](#_ENREF_3)] |
| Nod factor | type 1 | type 2 | [[3](#_ENREF_3)] |
| Competitiveness | higher | lower | [[2](#_ENREF_2), [4](#_ENREF_4)] |
| LPS and protein profiles | | | |
| LPS profile | type 1 | type 2 | CPAC 7 [[2](#_ENREF_2), [5](#_ENREF_5)]; CPAC 15 [[2](#_ENREF_2)] |
| Protein profile | type 1 | type 2 | CPAC 7 [[2](#_ENREF_2), [5](#_ENREF_5)]; CPAC 15 [[2](#_ENREF_2)] |
| Genetic characterization | | | |
| RAPD | type 1 | type 2 | [[6](#_ENREF_6)] |
| ERIC-PCR | type 1 | type 2 | CPAC 7 [[5](#_ENREF_5)]; CPAC 15 [[2](#_ENREF_2)] |
| REP-PCR | type 1 | type 2 | CPAC 7 [[5](#_ENREF_5), [7](#_ENREF_7)]; CPAC 15 [[7](#_ENREF_7), [8](#_ENREF_8)] |
| BOX-PCR | type 1 | type 2 | [[7](#_ENREF_7)] |
| RFLP-PCR |  |  |  |
| 16S-CfoI (=HhaI) | type I | type I | [[7](#_ENREF_7), [9](#_ENREF_9)] |
| 16S-MspI (=HpaII) | type I | type I | [[7](#_ENREF_7), [9](#_ENREF_9)] |
| 16S-HinfI | type I | type I | [[10](#_ENREF_10)] |
| 16S-DdeI | type I | type 2 | [[7](#_ENREF_7), [9](#_ENREF_9)] |
| IGS-HinfI | type I | type I | [[10](#_ENREF_10)] |
| IGS-CfoI (=HhaI) | type I | type I | [[10](#_ENREF_10)] |
| IGS-MboI | type I | type I | [[10](#_ENREF_10)] |
| IGS-RsaI | type I | type 2 | [[10](#_ENREF_10)] |
| IGS-MspI (=HpaII) | type I | type 2 | [[9](#_ENREF_9), [10](#_ENREF_10)] |
| IGS-DdeI | type I | type 2 | [[9](#_ENREF_9)] |
| IGS-HaeIII | type I | type 1 | [[9](#_ENREF_9)] |
| 23S-HaeIII | type I | type 2 | [[9](#_ENREF_9)] |
| 23S-HinfI | type I | type 2 | [[9](#_ENREF_9)] |
| 23S-CfoI (=HhaI) | type I | type 2 | [[9](#_ENREF_9)] |
| Gene sequencing |  |  |  |
| 16S rRNA | G-II.2.1 | G-II.2.3 | [[11](#_ENREF_11)] |
| MLSA group | GI-7 | GI-6 | [[12](#_ENREF_12)] |

## References

1. Boddey LH, Hungria M: **Phenotypic grouping of Brazilian *Bradyrhizobium* strains which nodulate soybean**. *Biol Fertil Soils* 1997, **25**:407-415.

2. Ferreira MC, Hungria M: **Recovery of soybean inoculants strains from uncropped soils in Brazil**. *Field Crops Res* 2002, **79**(2-3):139-152.

3. Hungria M, Nishi CYM, Cohn J, Stacey G: **Comparison between parental and variant soybean *Bradyrhizobium* strains with regard to the production of lipo-chitin nodulation signals, early stages of root infection, nodule occupancy, and N-2 fixation rates**. *Plant Soil* 1996, **186**:331-341.

4. Mendes I, Hungria M, Vargas M: **Establishment of *Bradyrhizobium japonicum* and *B. elkanii* strains in a Brazilian Cerrado oxisol**. *Biol Fertil Soils* 2004, **40**(1):28-35.

5. Santos MA, Vargas MAT, Hungria M: **Characterization of soybean *Bradyrhizobium* strains adapted to the Brazilian savannas**. *FEMS Microbiol Ecol* 1999, **30**(3):261-272.

6. Nishi CYM, Boddey LH, Vargas MAT, Hungria M: **Morphological, physiological and genetic characterization of two new *Bradyrhizobium* strains recently recommended as Brazilian commercial inoculants for soybean**. *Symbiosis* 1996, **20**(2):147-162.

7. Batista JSS, Hungria M, Barcellos FG, Ferreira MC, Mendes IC: **Variability in *Bradyrhizobium japonicum* and *B. elkanii* seven years after introduction of both the exotic microsymbiont and the soybean host in a Cerrados soil**. *Microbial Ecol* 2007, **53**:270-284.

8. Hungria M, Boddey LH, Santos MA, Vargas MAT: **Nitrogen fixation capacity and nodule occupancy by *Bradyrhizobium japonicum* and *B. elkanii* strains**. *Biol Fertil Soils* 1998, **27**:393-399.

9. Germano MG, Menna P, Mostasso FL, Hungria M: **RFLP analysis of the rRNA operon of a Brazilian collection of bradyrhizobial strains from 33 legume species**. *Int J Syst Evol Microbiol* 2006, **56**(1):217-229.

10. Alberton O, Kaschuk G, Hungria M: **Sampling effects on the assessment of genetic diversity of rhizobia associated with soybean and common bean**. *Soil Biology and Biochemistry* 2006, **38**(6):1298-1307.

11. Menna P, Hungria M, Barcellos FG, Bangel EV, Hess PN, Martinez-Romero E: **Molecular phylogeny based on the 16S rRNA gene of elite rhizobial strains used in Brazilian commercial inoculants**. *Syst Appl Microbiol* 2006, **29**(4):315-332.

12. Menna P, Pereira AA, Bangel EV, Hungria M: **rep-PCR of tropical rhizobia for strain fingerprinting, biodiversity appraisal and as a taxonomic and phylogenetic tool**. *Symbiosis* 2009, **48**:120-130.

**ABLE S6 Main features of the genomic islands (GIs) identified in *B. japonicum* CPAC 15 and *B. diazoefficiens* CPAC 7**

| **Genomic Island (GI)** | **Initial Locus** | **Final Locus** | **tRNA (Locus)** | **Size (kbp)** | **Relevant encoded proteins** |
| --- | --- | --- | --- | --- | --- |
| *B. japonicum*CPAC 15 | | | | | |
| 1 | BS07078 | BS02379 | tRNA-Pro (BS08686) | 345.14 | Heat Shock Proteins / Chaperonins, Osmoregulated perisplasmic glucan biosynthesis proteins |
| 2 | BS08758 | BS03391 | None | 74.58 | DNA methyltransferase, Type II restriction endonuclease |
| 3 | BS03503 | BS03552 | tRNA-Asn (BS08662) | 59.58 | Polysaccharide biosynthesis proteins, Chaperone |
| 4 | BS06030 | BS05956 | tRNA-Gln (BS08682) | 100.44 | Type I secretion system proteins, Type II DNA modification methyltransferase |
| 5 | BS05862 | BS08976 | None | 104.81 | Type I secretion system proteins |
| 6 | BS00460 | BS00491 | None | 38.26 | Type IV secretion system proteins, Nitrogen fixation regulation protein (*fixK* - BS00504) |
| 7 | BS00400 | BS00444 | None | 59.22 | Lipopolysaccharide biosynthesis proteins |
| 8 | BS01438 | BS01512 | tRNA-Met (BS08646) | 73.54 | Trehalose biosynthesis protein |
| 9* | BS08880 | BS07677 | None | 211.13 | Type IV secretion system proteins |
| 10 | BS00707 | BS00738 | None | 45.73 | Metal-resistance proteins |
| 11 | BS04636 | BS04640 | tRNA-Arg (BS08680) | 9.15 | - |
| 12 | BS07884 | BS07933 | tRNA-Met (BS08687) | 51.43 | Urease |
| SI | BS08328- BS07503 | BS08139- BS08640 | tRNA-Val (BS08685) | 698.37 | Symbiosis gene proteins |
| *B. diazoefficens* CPAC 7 | | | | | |
| 1 | BU02185 | BU02214 | tRNA-Gly (BU08213) | 58.82 | Type I secretion system exported protein |
| 2 | BU03452 | BU03353 | tRNA-Lys (BU08225) | 117.91 | Formate hydrogenase proteins, Type I secretion system proteins |
| 3 | BU01465 | BU01331 | tRNA-Met (BU08217) | 147.49 | Type I secretion system proteins, Thioredoxin peroxidase, Molybdenum ABC-transporter, Uptake hydrogenase, Restriction system protein, Copper resistance proteins, Nitrate/nitrite transporter |
| 4 | BU00957 | BU00910 | tRNA-Gln (BU08210) | 80.7 | Restriction system proteins, DNA methylase |
| 5 | BU08394 | BU08383 | tRNA-Thr (BU08251) | 41.27 | Type I restriction-modification system proteins |
| 6 | BU07809 | BU05145 | tRNA-Met (BU08240) | 65.21 | Type I secretion system proteins |
| 7 | BU06090 | BU06106 | tRNA-Arg (BU08235) | 24.31 |  |
| 8* | BU07124 - BU07197 | BU06189 - BU08196 | None | 111.67 | Type IV secretion system proteins |
| 9 | BU02350 | BU02359 | tRNA-Thr (BU08219) | 11.31 | **-** |
| 10 | BU04111 | BU08388 | tRNA-Ser (BU08226) | 13.49 | **-** |
| 11 | BU03087 | BU03057 | None | 30.8 | Lipopolysaccharide biosynthesis proteins |
| 12 | BU05564 | BU05570 | tRNA-His (BU08238) | 19.79 | **-** |
| 13 | BU07430 | BU07447 | tRNA-Ser (BU08252) | 21.61 | Nitrous oxide reductase component proteins |
| 14 | BU05375 | BU05385 | None | 14.02 | **-** |
| 15 | BU06629 | BU06542 | tRNA-Leu (BU08245) | 78.77 | Heat Shock Proteins |
| SI** | BU02649 | BU07755 | tRNA-Val (BU08255) | 687.93 | Symbiosis gene proteins |

* Symbiosis island locus C as defined by Kaneko et al. (2011).

** Symbiosis island [locus A as defined by Kaneko et al. (2011)].

Kaneko T, Maita H, Hirakawa H, Uchiike N, Minamisawa K, Watanabe A, Sato S: **Complete genome sequence of the soybean symbiont *Bradyrhizobium japonicum* strain USDA6^T^**. *Genes* 2011, **2**(4):763-787.

**Table S7 Secondary metabolites genes or gene clusters of *B. japonicum* CPAC 15 and *B. diazoefficiens* strain CPAC 7 defined by antiSMASH (Antibiotics & Secondary Metabolite Analysis Shell)** (Medena et al., 2011)

|  | **ORFs in** | |
| --- | --- | --- |
| **Cluster type** | **CPAC 7** | **CPAC 15** |
| Terpene | BU00557 - BU00553 | BS06370 - BS06366 |
| Bacteriocin | BU04098 - BU04097 | BS03326 - BS03327 |
| Terpene | BU07070 | BS03170 |
| Non ribosomal peptide | BU03951 - BU03949 | BS07445 - BS07443 |
| Polyketide |  | BS00720 |

Medema MH, Blin K, Cimermancic P, de Jager V, Zakrzewski P, Fischbach MA, Weber T, Takano E, Breitling R: **antiSMASH: rapid identification, annotation and analysis of secondary metabolite biosynthesis gene clusters in bacterial and fungal genome sequences**. *Nucleic Acids Res* 2011, **39**(suppl. 2):W339-W346
