# Supplementary material for: Comparative genomics of Bradyrhizobium japonicum CPAC 15 and Bradyrhizobium diazoefficiens CPAC 7: elite model strains for understanding symbiotic performance with soybean
Source: BMC Genomics. 2014 Jun 3;15(1):420. doi: 10.1186/1471-2164-15-420 (PMC4070871; doi:10.1186/1471-2164-15-420)
Supplement: Supplementary file 2 — Additional file 2: Supplementary figures. (DOCX 1 MB) [file 12864_2014_6142_MOESM2_ESM.docx]

**Figure S1 Percentage of ORFs of *B. japonicum* CPAC 15 and *B. diazoefficiens* CPAC 7 in each KEGG functional class.**


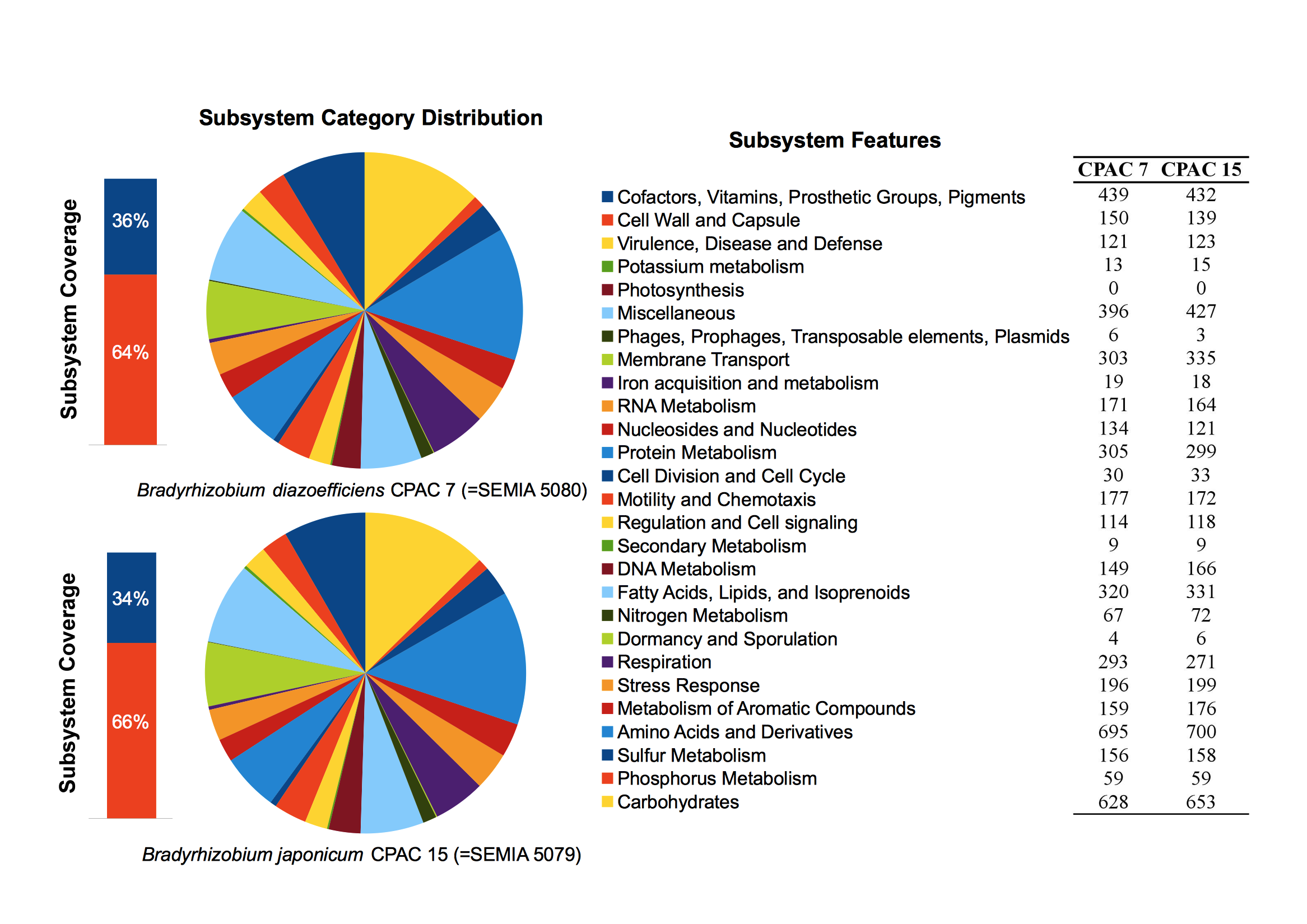


**Figure S2. Functional characterization of the genomes of *B. japonicum* strain CPAC 15 and *B. diazoefficiens* strain CPAC 7 according to MG-RAST.**


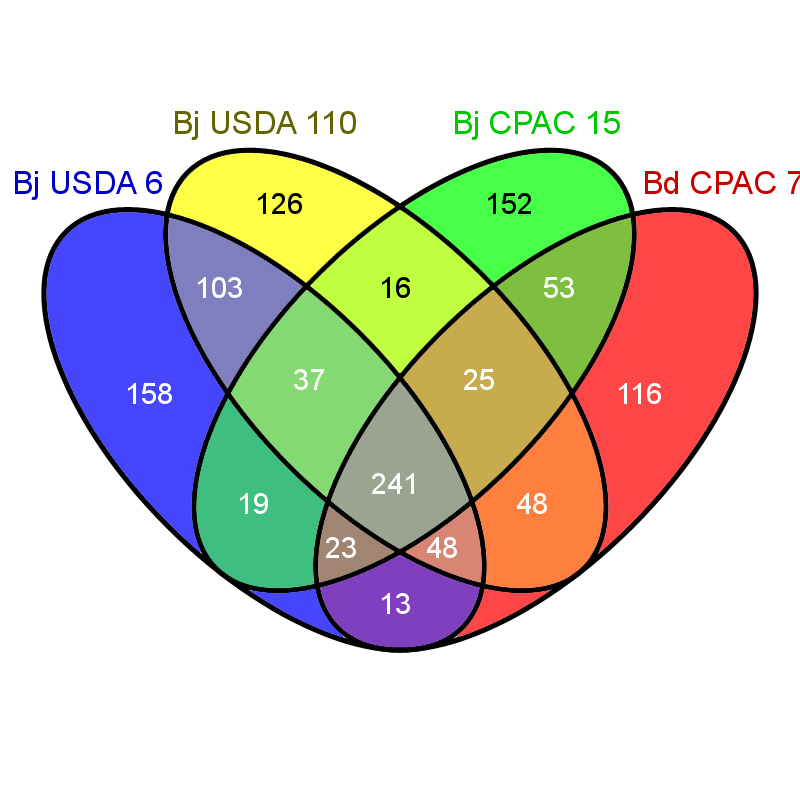


Figure S3. Venn diagram depicting overlaps and differences in orthologous genes of the symbiosis islands of *B. japonicum* strains USDA 6^T^ and CPAC 15 and *B. diazoefficiens* strains USDA 110^T^ and CPAC 7.


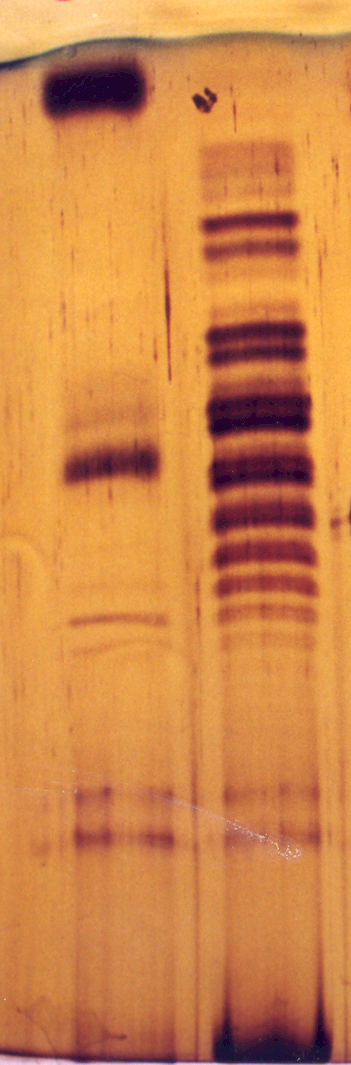


1. **(B)**

**Figure S4 Lipopolysaccharide profiles.** **A.** *B. diazoefficiens* CPAC 7. **B.** *B. japonicum* CPAC 15.


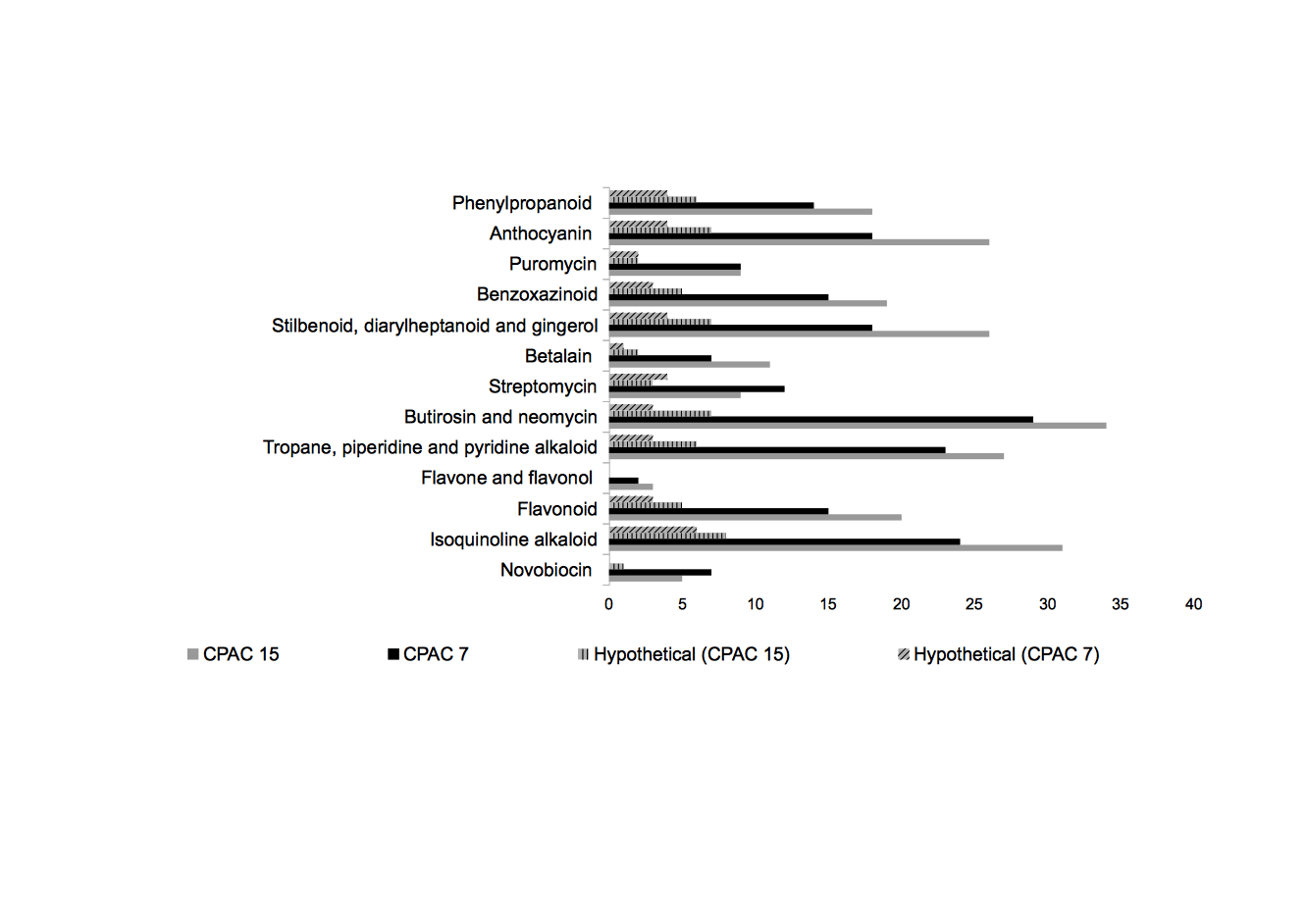


**Figure S5 ORFs related to the biosynthesis of secondary metabolites in *B. japonicum* CPAC 15 and *B. diazoefficiens* CPAC 7.**
